# Synchrotron Scattering Methods for Nanomaterials and Soft Matter Research

**DOI:** 10.3390/ma13030752

**Published:** 2020-02-06

**Authors:** Theyencheri Narayanan, Oleg Konovalov

**Affiliations:** European Synchrotron Radiation Facility (ESRF), F-38043 Grenoble, France; konovalo@esrf.fr

**Keywords:** X-ray scattering, nanomaterials, soft matter, SAXS, GISAXS, XPCS, XRR, GID, USAXS

## Abstract

This article aims to provide an overview of broad range of applications of synchrotron scattering methods in the investigation of nanoscale materials. These scattering techniques allow the elucidation of the structure and dynamics of nanomaterials from sub-nm to micron size scales and down to sub-millisecond time ranges both in bulk and at interfaces. A major advantage of scattering methods is that they provide the ensemble averaged information under in situ and operando conditions. As a result, they are complementary to various imaging techniques which reveal more local information. Scattering methods are particularly suitable for probing buried structures that are difficult to image. Although, many qualitative features can be directly extracted from scattering data, derivation of detailed structural and dynamical information requires quantitative modeling. The fourth-generation synchrotron sources open new possibilities for investigating these complex systems by exploiting the enhanced brightness and coherence properties of X-rays.

## 1. Introduction

A large fraction of modern engineering materials are based on nanomaterials, composed of nanometer sized building blocks, and their organization at multiple scales. Examples include nanoparticles, nanocomposites, quantum dots, fullerenes, nanofoams, nanoporous materials, and so on [1]. Soft matter systems, such as colloids, polymers, surfactants, liquid crystals, biological macromolecules, etc., are another class of nanomaterials in which organization at the nanoscale often determines their macroscopic properties [2,3]. Often these building blocks self-assemble through competing interactions resulting in an hierarchical structure which is key to their bottom-up fabrication. Many fascinating macroscopic features exhibited by these systems are direct results of such hierarchical structural organization.

Many advanced materials are two-dimensional (2D) in extent and confined in one direction to nanometer scale. These two-dimensional materials (2DM) have great promise for applications in emerging technologies such as organic solar cells, light-emitting diodes, field effect transistors, topological insulators, biosensors, biomembranes, etc. 2DM can be formed on hard solids, soft, and liquid substrates, of which latter media are particularly interesting due to their flexibility. Building blocks (macromolecules or nanoparticles) that are trapped in one dimension have a higher mobility (both translational and rotational) in the plane, owing to weak interactions with the support and thermal fluctuations. The high mobility in combination with relatively weak interactions lead to self-assembly of these building blocks into a perfect 2D order, thereby enhancing their desired physical properties.

To advance the design of nanomaterials, powerful tools for observation and quantification of their structures are required [4]. X-ray scattering methods are well suited for the structural investigation of these materials both in bulk and at the interfaces. The scattering contrast originates from the spatial fluctuations of the electron density that systematically varies with the atomic number of constituent elements. Small-angle X-ray scattering (SAXS) provides structural resolution of the order of 1–100 nm for investigations in the bulk [5]. Wide-angle X-ray scattering (WAXS) pushes the range down to smaller scales, enabling structural elucidation at atomic and molecular ranges in systems lacking perfect crystalline order, albeit at lower resolution compared to crystallography. In the case of 2DM, relatively weak scattering signal from the nanometer-thick specimen needs to be discriminated from the bulk scattering of the substrate material. This is achieved by measurements carried out at grazing incidence (GI) geometry as opposed to bulk transmission configuration used in SAXS and WAXS. The analogous surface sensitive techniques are grazing incidence SAXS and WAXS (GISAXS and GIWAXS, respectively). The X-ray reflectivity (XRR) is the most widely used surface scattering technique that probes the density profile across an interface [6]. GISAXS and GIWAXS elucidate in-plane structural organization and provide complementary information to that obtained by XRR. Traditionally, GISAXS and GIWAXS have been used to study noncrystalline organization of macromolecules and nanoscale objects. With the emergence of nanoscience, GISAXS is routinely employed for studying crystalline organization of nanoparticles and other self-assembled systems.

Advances in instrumentation have allowed SAXS to reach length scales up to μm and above by ultra small-angle X-ray scattering (USAXS) method, thereby providing a good overlap with a broad range of imaging techniques [3,7]. The higher angular resolution of the USAXS setup is also important for the investigation of long-range periodic order in self-assembled nanomaterials and soft matter systems [8]. The high brilliance offered by modern synchrotrons enables investigations of time-dependent processes and transient dynamics in real-time by X-ray scattering methods [3]. In addition, the partial coherence of the synchrotron X-ray beam can be exploited to probe the equilibrium dynamics in appropriate systems by X-ray photon correlation spectroscopy (XPCS) [9], which is X-ray analog of dynamic light scattering (DLS). The 2D speckle pattern generated when a noncrystalline specimen is illuminated by a coherent X-ray beam also permits lensless imaging, which is known as coherent X-ray diffractive imaging (CDI or CXDI) [10].

This review focuses on some specific synchrotron X-ray scattering experiments, which investigated nanomaterials and soft matter in bulk and at interfaces primarily carried out at the ESRF. The focus is mainly on in situ and in operando studies. As a result, the literature covered is not exhaustive but presents representative examples from recent years. Moreover, the high X-ray contrast of many nanomaterials would allow their structural investigations using laboratory X-ray instruments and such examples are not included in this review.

## 2. X-ray Scattering Methods

This section outlines the basic principles of X-ray scattering methods used in the examples presented in this article. The main purpose is to define the notations and then show typical information that can be derived.

### 2.1. Small-Angle X-ray Scattering

**Figure 1:**
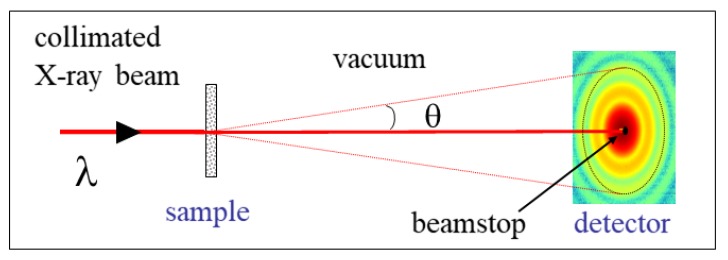
Schematic representation of a SAXS experiment set-up showing the incident, scattered, and transmitted beams; the 2D detector; and the beamstop.

The basic formalism of small-angle scattering method is similar for light, neutrons, and X-rays, but the main difference is the interaction between the incident radiation and the scattering medium. The scattering contrast in the case of X-rays originates from the spatial fluctuations of electron density, and it is given by the difference in the scattering length density (SLD) of the structural units and the surrounding medium [5]. Figure 1 shows the scattering geometry of a typical SAXS experiment set-up. A highly collimated and monochromatic X-ray beam of wavelength, λ, traverses a sample and the scattered intensity in the forward direction is recorded by a 2D detector, i.e., the number of photons scattered as a function of the scattering angle, θ. The transmitted X-ray beam is blocked by a beamstop in front of the detector, and the flight paths before and after the sample are in vacuum to avoid absorption and scattering by air. Scattering at small angles is fully elastic and therefore the magnitudes of incident and scattered wave vectors ($k_{i}$ and $k_{s}$, respectively) are equal to 2π/λ. The scattering vector, **q** = **k**${}_{s}$−**k**${}_{i}$, and its magnitude (*q*) is given by

(1) $$q=\frac{4\pi }{\lambda }sin\left(\theta /2\right)$$

The nominal size scale probed by a scattering experiment is given by the range of 2π/q covered. The quantity that can be compared in different measurements is the number of photons scattered into unit solid angle of the detector normalized by the incident flux (photons per second per unit area) and sample thickness, and it is called the differential scattering cross section per unit volume (dΣ/dΩ). Indeed, the measured intensity also needs to be normalized by the sample transmission (for absorption losses in the illuminated volume) and detector efficiency [5]. The resulting quantity (dΣ/dΩ) contains information on the structure and the interactions among the scattering objects in the illuminated volume over the range of *q* spanned by the scattering experiment. It is expressed in units of reciprocal length per solid angle (m${}^{-1}$ sterad${}^{-1}$) and in practice denoted by I(q). For a given λ, depending upon the sample-detector distance (i.e., θ), different size scales are probed. Typically, SAXS probes sizes from approximately 1 nm to above 100 nm, WAXS elucidates sizes smaller than 1 nm down to the atomic scale, and USAXS explores sizes larger than 100 nm up to several microns. The scale is continuous with overlapping ranges between SAXS and WAXS, and SAXS and USAXS techniques. The high brilliance of synchrotron X-ray enables time-dependent measurements down to the millisecond range and investigations of kinetic processes, which will be described in the subsequent Sections.

### 2.2. X-ray Photon Correlation Spectroscopy

The experiment set-up for multispeckle XPCS measurement is similar to SAXS and WAXS, but the coherent fraction of the incident beam is selected by small slits or apertures (10–20 μm). The measured scattering patterns then display speckles which represent the diffraction-limited structure function of the scattering units in the medium. The measurement involves recording of a sequence of 2D speckle patterns with exposure and lag time between frames much shorter than the typical relaxation times probed within the sample. From the temporal fluctuations of the speckles, the intensity–intensity autocorrelation function, $g_{2}\left(q,t\right)$, is calculated pixel by pixel. In multispeckle XPCS, $g_{2}\left(q,t\right)$s corresponding to the same *q* can be averaged to obtain the ensemble averaged $g_{2}\left(q,t\right)$.

(2) $$g_{2}\left(q,t\right)=\frac{\langle I\left(q,t_{0}\right)I\left(q,t_{0}+t\right)\rangle }{{\langle I(q,t_{0})\rangle }^{2}}$$

with I(q,t) being the scattered intensity measured at a given *q* at time *t* and <·> denotes the time average. The $g_{2}\left(q,t\right)$ is related to the corresponding electric field-field autocorrelation function, $g_{1}\left(q,t\right)$, via the Siegert-relation,

(3) $$g_{2}\left(q,t\right)=1+g_{0}{\left|g_{1}\left(q,t\right)\right|}^{2}$$

where $g_{0}$ is the speckle contrast, which depends on not only the coherence properties of the incoming X-ray beam but also the angular resolution of the scattering setup. In the ideal case of a perfect coherent beam and speckle size larger than the detector pixel size, $g_{0}≃1$. However, due to the limited coherence of the synchrotron beam and detector resolution, this factor is usually much smaller than one. The underlying dynamics of the system is manifested in the $g_{1}\left(q,t\right)$.

### 2.3. Surface and Interface X-ray Scattering

**Figure 2:**
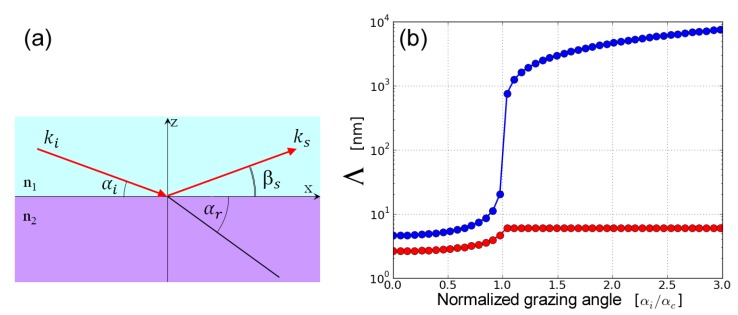
(**a**) Reflection and refraction of an incident X-ray beam at the plane of incidence. (**b**) The penetration depth in water depending on the incident grazing angle (blue curve). Red curve shows the effective penetration depth depending on the outgoing grazing angle with the incident angle fixed at 65% of the critical angle.

In the grazing incidence geometry, the grazing angles of incidence ($\alpha_{i}$) and refraction ($\alpha_{r}$) are related by the Snell’s law $cos\left(\alpha_{i}\right)/cos\left(\alpha_{r}\right)=n$, with *n* the refractive index, as shown in Figure 2a. The refractive index, n=1−δ−iβ, in the X-ray region it is slightly below one, and for typical soft matter systems, the real (δ) and imaginary (β) parts of the decrement are of the order of $10^{-6}$ and $10^{-8}$, respectively [11]. As a result, there is a critical angle ($\alpha_{c}$) below which there is no refraction and the beam is specularly reflected. From the Snell’s law, the critical angle, $\alpha_{c}={\left(2\delta \right)}^{1/2}$, as $cos(\alpha_{r})≃1$. Below $\alpha_{c}$, the penetration of X-rays in the medium is very small and only an evanescent wave that decays exponentially over several nanometers is present below the surface [11]. The penetration depth of this wave, Λ, defined as the distance over which the intensity is attenuated by a factor *e* can be calculated from the imaginary part of the grazing beam wave number ${\tilde{k}}_{i}=\sqrt{n^{2}-cos^{2}\left(\alpha_{i}\right)}$

(4) $$\Lambda =-\frac{1}{2k_{0}Im\left({\tilde{k}}_{i}\right)}$$

where $k_{0}=2\pi /\lambda$. Figure 2b (blue curve) shows the variation of Λ with normalized grazing angle of X-rays impinging on water surface. Λ does not change much up to 80% of $\alpha_{i}$ and it is ~5 nm but exponentially grows above $\alpha_{c}$. The small and adjustable penetration of X-rays at grazing incidence is a key feature for enhancing the surface scattering as compared to the bulk contribution. Although, the grazing angle defines the maximum penetration depth, the effective penetration depth, $\Lambda_{eff}$, depends also on the scattering angle $\beta_{s}$ as follows,

(5) $$\Lambda_{eff}=-\frac{1}{2k_{0}Im\left({\tilde{k}}_{i}+{\tilde{k}}_{s}\right)}$$

where ${\tilde{k}}_{s}=\sqrt{n^{2}-cos^{2}\left(\beta_{s}\right)}$. The red line in Figure 2b shows $\Lambda_{eff}$ for water at different $\beta_{s}$ and $\alpha_{i}/\alpha_{c}=0.65$. Notice that for $\beta_{s}<\alpha_{c}$, the probing depth of an interface is smaller than that at $\alpha_{c}$ and at larger exit angles. In conclusion, to be surface-sensitive, the measurements have to be performed below the critical angle of total reflection that is typically about a few milliradians. Therefore, GISAXS requires a small vertical beam size (VBS) to match the beam footprint (FP) with the sample size, $FP=VBS/sin(\alpha_{i})$.

**Figure 3:**
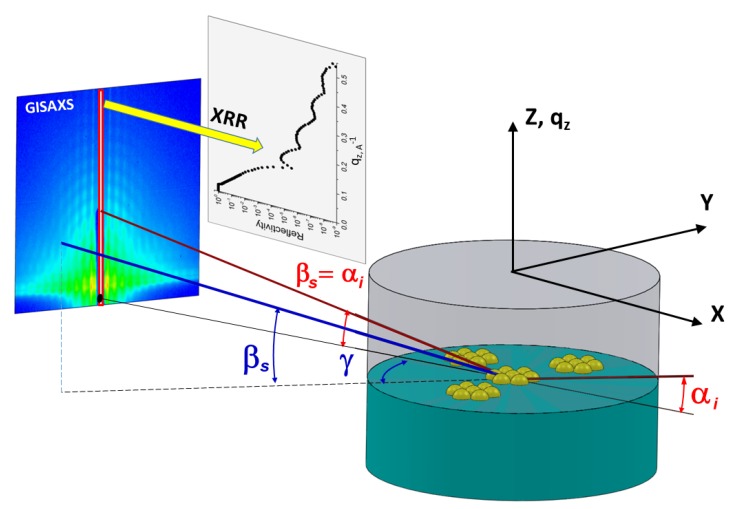
Schematic illustration of the scattering geometry at an interface. Here, $\alpha_{i}$, $\beta_{s}$, and γ are incidence, exit, and in-plane angles, respectively. XRR ($\beta_{s}=\alpha_{i}$) probes the density profile along the z direction while the off-specular scattering (GISAXS or GIWAXS) elucidates lateral structural organization at the interface.

There are several surface sensitive X-ray scattering methods based on the measurement geometry, the variation of incoming angle ($\alpha_{i}$), and scattering angle described by the out-of-plane ($\beta_{s}$) and in-plane (γ) angles illustrated in Figure 3. Here, in-plane angle is in the plane of the interface and out-of-plane angle is in the plane perpendicular to the interface. Correspondingly, the scattering vector is composed of three components shown in Figure 3,

(6) $$\left\{\begin{array}{c} q_{x}\\ q_{y}\\ q_{z} \end{array}\right\}=\frac{2\pi }{\lambda }\left\{\begin{array}{c} cos\beta_{s}\cdot cos\gamma -cos\alpha_{i}\\ cos\beta_{s}\cdot sin\gamma \\ sin\alpha_{i}+sin\beta_{s} \end{array}\right\}$$

In the case of XRR, $\alpha_{i}$ and $\beta_{s}$ are changed simultaneously (i.e., $\alpha_{i}=\beta_{s}$), and γ=0, keeping the scattering vector normal to the interface. The XRR derives the average SLD profile of the interface along its normal, and the corresponding continuous electron density profile is interpreted in terms of thickness and density of constituting layers, and associated interfacial roughness. GISAXS is analogous to SAXS on a surface and applied to study particle geometry, size distribution, and spatial correlations at the interface. When crystalline features of 2DM are investigated, GIWAXS is also referred to as grazing incidence X-ray diffraction (GID or GIXD). The geometry of these grazing incidence scattering methods is $\alpha_{i}<\alpha_{c}$, γ≥0, $\beta_{s}\geq 0$. Analogous to WAXS, GIWAXS is applied to study the structure of 2DM at the intermolecular and interatomic scales. GID elucidates the structural details of 2D crystals such as lattice parameter, molecular structure, tilt angle, and tilt azimuth of rod-like molecules, as well as in-plane correlation lengths. In the absence of crystalline order, GIWAXS provides information about the short-range fluctuations on a sample surface, e.g., capillary waves at a liquid surface [6].

Another surface sensitive X-ray technique is grazing incidence X-ray fluorescence (GIXF), which measures the florescence signal as a function of grazing angle by an energy-resolved detector placed perpendicular to the X-ray beam and installed either above the sample surface (corresponding to $\alpha_{i}<\alpha_{c}$, γ = 90°) or laterally close to the sample surface. GIXF method brings additional information about depth-resolved elemental distribution profile [12].

### 2.4. Instrumentation for Bulk and Interface Scattering

**Figure 4:**
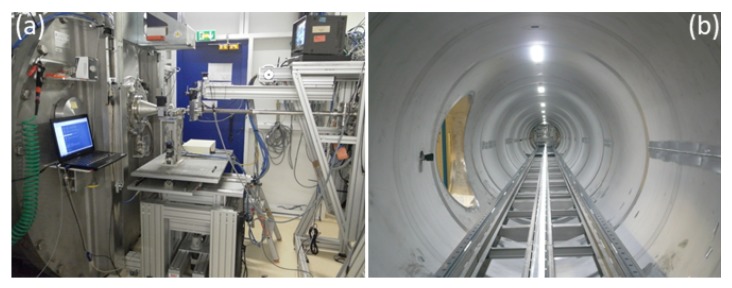
Experiment station at ID02 beamline at the ESRF. (**a**) The photo displays the sample table and stages, incident side telescopic tube, and the entrance cone of the detector tube. (**b**) Inside view of the 34 m long and 2 m diameter detector tube showing the detector carriage at the end.

Beamline ID02 at the ESRF is a multipurpose instrument optimized for time-resolved small-angle X-ray scattering (TR-SAXS) with high angular resolution [13]. Figure 4 depicts the experiment set-up with 34 m long evacuated detector flight tube. The sample–detector distance can be varied from about 1 m corresponding to the conventional SAXS range to 31 m spanning the USAXS region. In combination with WAXS and USAXS, the instrument covers a broad range of *q*, from 10${}^{-3}$ nm${}^{-1}$ to approximately 60 nm${}^{-1}$, corresponding to a nominal real space dimension from about 6 μm down to 0.1 nm. Access to such a broad range of size scales is useful for elucidating the hierarchical structure in many complex systems. In time-dependent studies, SAXS, WAXS, and USAXS measurements can be performed with millisecond time resolution. All detectors are 2D, which, in the case of isotropic scattering, improves the intensity statistics by azimuthal averaging. In addition, the transmitted primary beam intensity is simultaneously recorded by a point detector embedded in the beamstop and thereby enabling precise normalization of measured scattered intensities to dΣ/dΩ. A variety of sample environments enable in situ and kinetic investigations [13]. By strongly collimating, a nearly coherent X-ray beam can be obtained that enables XPCS measurements in the SAXS and USAXS range.

**Figure 5:**
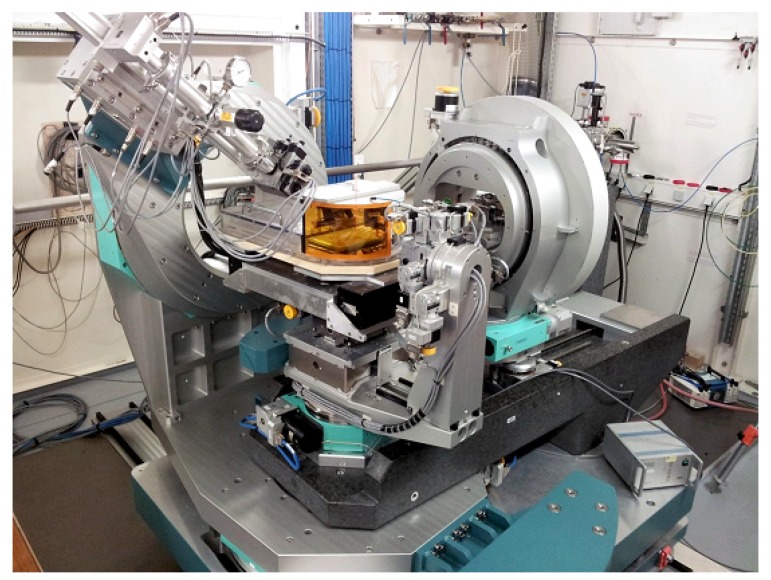
Surface and Interface scattering setup at ID10 beamline at the ESRF. The photo displays the 2 + 2 circle diffractometer, the double crystal beam deflector, Langmuir trough, and the detector arm.

The ID10 beamline at the ESRF hosts a multipurpose instrument for the study of liquid and solid interfaces, combining GID, XRR, and GISAXS techniques in a single set-up. The surface scattering instrument comprises a beam deflector stage for experiments on liquid surfaces and a multipurpose 2 + 2 circle diffractometer as displayed in Figure 5. The double crystal deflector rotates the X-ray beam around a fixed point on the liquid surface so that the sample does not move in space during a measurement. High precision studies on liquid surfaces are possible thanks to the Langmuir through mounted on an active antivibration table. The diffractometer has a detector arm carrying up to three detectors and sample stages for both horizontal and vertical scattering geometries. Several techniques, like GISAXS, GIWAXS, and GIXF, can be performed simultaneously. With these techniques, length scales from sub-nm to 100 nm, in some cases even up to 1000 nm, can be probed. This allows time-resolved investigations of self-organization processes at surfaces, interfaces, and in thin films. High-resolution studies are possible in both scattering geometries via the use of analyzer crystal stages in different orientations. In addition, this instrument can be used for XPCS in GI configuration (GIXPCS) to study interface dynamics. In situ and often simultaneous measurements of scattering signals on different length scales in reciprocal space require a complex sample environment to control different physical parameters, such as temperature, pressure, solvent partial pressure, evaporation rate, etc., e.g., such a set-up has been developed for real-time GISAXS, GIWAXS, and XRR measurements on organic photovoltaics films [14].

The second experiment station at ID10 beamline is optimized for coherent X-ray scattering. The XPCS can be performed in both SAXS and WAXS configurations down to the sub-millisecond time range. This setup also permits full-field imaging of three-dimensional (3D) specimen by CDI in the SAXS geometry combined with tomographic reconstruction.

## 3. Investigations of Structure and Dynamics in Bulk Samples

This section presents selected examples of static and time-dependent studies of nanomaterials and soft matter by X-ray scattering predominantly in their suspensions. The list is certainly not exhaustive but illustrates certain unique information that can be derived from scattering experiments under appropriate thermodynamic conditions.

### 3.1. Equilibrium Nanostructure and Interactions

Here, some representative examples in which SAXS played an important role in elucidating the nanostructure and interactions are described. Traditionally, SAXS and related methods have been widely employed for the characterization of particulate systems such as colloids [5], polymers [15], surfactant micelles [16] and vesicles [17], lipid membranes [18] and particles (e.g., cubosomes and hexosomes) [19], proteins [20], etc. SAXS and GISAXS methods have been extensively used in nanoparticle research in particular for in situ studies [21,22]. In dilute samples, the main structural features derived are average size, polydispersity, shape and morphology of particles, and the internal density distribution [5,22]. The high brilliance of synchrotron X-rays has enabled studies of extremely dilute systems, such as aerosol suspensions [23], dusty plasmas [24], etc., and allowed obtaining the mean size and size distribution of primary particles as well as their aggregates and agglomerates. In sterically stabilized colloids, a systematic variation of surface grafts and their influence on the colloidal stability has been probed by USAXS [25], and, surprisingly, the shorter grafts were found to provide a better stability against the salting-out effect [26]. In concentrated systems, the interparticle interactions are significant, and the measured SAXS intensity becomes dominant of the structure factor, S(q), of interactions [5,22,27]. A quantitative analysis of S(q) provides the strength and range of the potential of mean force between the particles. Highly concentrated samples of uniform particles form a variety of ordered states such as colloidal crystals [8] or lyotropic phases in the case of anisotropic particles [28] and high-resolution SAXS revealed their structure and long-range order within.

Colloidal systems also turn into gels and glasses depending on the concentration and interactions, and SAXS has been used to probe the underlying long-range and short-range interactions [27,29,30]. A similar approach has been employed to unravel the combined effects of ionic strength, temperature, and pressure on protein–protein interaction potential and the phase behavior in dense lysozyme solutions [31]. A recent SAXS investigation probed the evolution of protein–protein interactions and liquid–liquid phase separation induced by trivalent salts and temperature in concentrated bovine serum albumin (BSA) solutions [32]. Proteins immobilized on polyelectrolyte brushes is another topic studied by SAXS, which enabled quantitative estimation of the concentration and location of adsorbed proteins within the brush layer [33,34]. SAXS can be used for easy screening of different micellar morphologies in interpolyelectrolyte complexes of miktoarm star polymers and diblock copolymer when the soluble arm number is systematically varied [35]. The mesoscopic scale structural complexity in room temperature ionic liquids (RTIL) was elucidated by SAXS and found that an intermediate range order appears to drive their peculiar properties [36].

**Figure 6:**
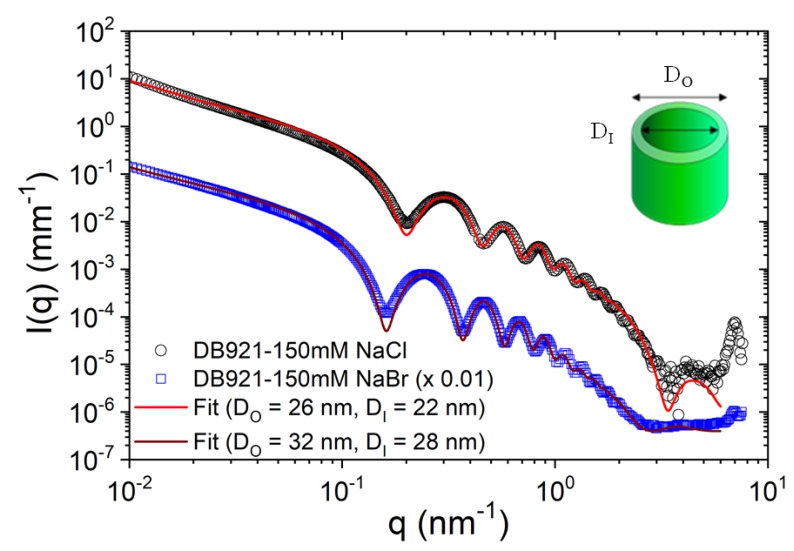
Azimuthally averaged SAXS profiles of nanotubes formed by a DNA minor groove-binding ligand DB921 in aqueous solutions containing halide salts. Modeling of the data provides the mean inner and outer diameters ($D_{I}$ and $D_{O}$, respectively) shown in the legend and polydispersity (∼0.05). Mean length of the tubes is many microns which is not covered by the *q* range of the measurement. The diameter and wall thickness of the tubes are larger in NaBr-containing solution as compared to that in NaCl solution. The peaks at higher *q* corresponds to the lateral packing of DB921 along the tube walls. Adapted from R. Mizuta et al. [39] ©the Royal Society of Chemistry 2018.

The broad range of size scales accessible by combined SAXS, WAXS, and USAXS is particularly suitable for elucidating the hierarchical supramolecular organization in a variety of self-assembled systems [3]. For instance, a combination of SAXS and USAXS elucidated the multiscale morphology in a prototypical photovoltaics (OPV) thin film consisting poly(3-hexylthiophene) (P3HT) and [6,6]-phenyl-C61-butyric acid methyl ester (PCBM) [37]. Combined SAXS and WAXS revealed the hierarchical morphology of molten and semicrystalline vitrimers [38]. A spectacular case is the hierarchical organization of certain amphiphilic molecules to form well-defined nanotubes [39,40] and microtubes [41], which can be unraveled by SAXS. For example, the nanotubes formed by some amphiphilic peptides such as amyloid β-peptide, which display multiple structural levels from the molecular scale up to the long range ordering of nanotubes [3]. Figure 6 depicts the structural features of a suspension of nanotubes formed by an heterocyclic ligand DB921 [39]. In this class of systems, the competing hydrophobic and electrostatic interactions lead to a variety of self-assembly pathways to form helical ribbons [39,42] and their closed conformation such as nanotubes [39,40].

Small-angle neutron scattering (SANS) and SAXS methods have been widely used for the elucidation of the morphology and internal organization of liposomes and other lipid nanoparticles loaded with drugs [19,43]. A key advantage is that measurements can be carried out in the same conditions of the pharmaceutical formulations. More recently, unilamellar vesicles with inclusions such as peptides, DNA, cholesterol, etc. have been studied with emphasis on locating the inclusions within the membrane [44,45,46]. The interaction between unilamellar phospholipid vesicles and antimicrobial peptides was probed very quantitatively using SAXS and contrast variation SANS [45]. In particular, this work clearly demonstrated the asymmetric distribution of peptides along the outer leaflet of the membrane that locally modifies the packing of the lipid tails. Significant changes in the lipid bilayer structure occurred, only beyond the physiologically relevant peptide/lipid ratios, supporting the interfacial activity scenario. A combination of USAXS, SAXS, and contrast variation SANS allowed the determination of the ultrastructure of *Escherichia coli* bacterial cell membrane [47] in vivo, thereby demonstrating the potential for monitoring the action of antimicrobial peptide on real cell membranes. SAXS was used to probe quantitatively the effect of additives aescin and ibuprofen on the structural parameters of a model phospholipid (DMPC) membrane and temperature-dependent phase transitions depending on the quantity of these additives [48]. The presence of these compounds are visible on the structure at different length scales ranging from the global morphology to inner membrane interactions. SAXS studies enabled the identification of structural features underlying the efficacity of microemulsion-based drug formulations [49] and lipid-based nanoformulatons [50]. The structure of polymeric drug delivery systems, especially the change in the inner structure upon drug encapsulation, was probed by anomalous SAXS [51]. Using polyethylene glycol (PEG) and trehalose, combined effects of osmotic pressure and hydrostatic pressure on the interaction between DMPC membranes and their topology were studied by SAXS [52].

The phase behavior of anisotropic colloids is very sensitive to applied fields such as electric or magnetic fields [53,54,55]. A field-induced isotropic to nematic transition can be observed in these suspensions. Orientation ordering of spindle-shaped hematite particles in a magnetic field was studied by combined SAXS and WAXS [55]. This work showed that the magnetic and orientation order parameters of magnetic single-domain nanospindles can be described by an oriented ellipsoid with the easy axis of magnetization lying in the equatorial plane of the particle. Texture analysis of the WAXS data further confirmed that the magnetic easy axis is located in the basal plane of the hematite crystal lattice [55]. The field-induced orientation of hematite particles can be used to probe the viscoelastic response of a gel such as that formed by poly(N-isopropylacrylamide) (PNIPAM) and correlate with the microrheological parameters [53]. With increasing elasticity of the gel, the transition to the nematic order occurred at progressively large value of the magnetic field. TR-SAXS was used to follow the rotational dynamics of anisotropic magnetic particles (e.g., hexaferrite platelets) in an alternating magnetic field [54] and explore the magneto-optical properties of the system.

The structural colors in both natural and synthetic systems have been the subject of investigation by SAXS [56,57]. These natural colors, such as in bird feathers, butterfly wings, insect scales, etc., originate purely from the underlying microstructure or biophotonic morphology developed by the phase separation of polymerizing β-keratin. The microstructure can be tuned continuously by the extent of phase separation and the measured structural colors can be analyzed in terms of one dimensional correlation functions [57]. Synthetic systems consisting of chameleon-like elastomers formed by the self-assembly of linear–bottlebrush–linear triblock copolymers display molecularly encoded strain-adaptive stiffening and coloration [58]. In these systems, the microphase separation of the architecturally distinct blocks results in physically cross-linked networks which can be explored by SAXS. The polymerization-induced self-assembly is a powerful approach for the synthesis of a range of block copolymer morphologies such as spheres, worms, vesicles, etc. [59]. SAXS has been used to follow the evolution of these morphologies and gain insight into their formation mechanism depending on the reaction conditions [59]. It is possible to encapsulate large amounts of nanoparticles in these large block copolymer vesicles during the synthesis and trigger their release over slower time scales by temperature or pH change [60]. SAXS studies showed that at lower loading densities, the complete release of particles is associated with a block copolymer vesicle to micelle transition, whereas at higher loading, the release is via perforations on the vesicles and remaining particles stabilize the vesicle structure [60].

The availability of extremely brilliant sources and high-performance detectors will improve the detection capability of SAXS and allied techniques, thereby enabling investigations of broader size scales and weaker structural features, which cannot be resolved by direct imaging methods [13]. Indeed, an appropriate sample environment is essential for performing an advanced in situ scattering experiment [61].

### 3.2. Probing the Pathways of Self-Assembly

**Figure 7:**
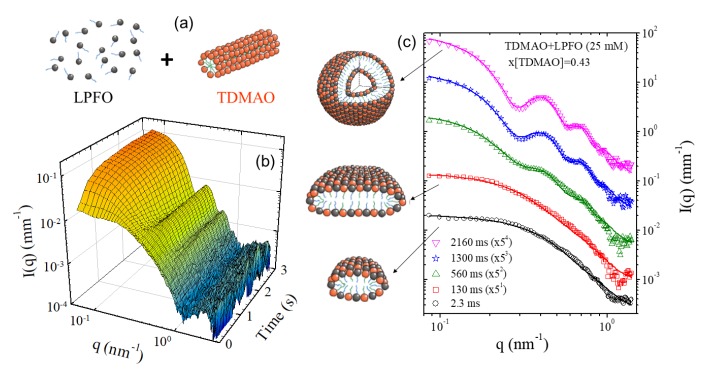
Self-assembly of unilamellar vesicles upon rapid mixing of 25 mM solutions of anionic lithium perfluorooctanoate (LPFO) surfactant and zwitterionic tetradecyldimethylamine oxide (TDMAO) micelles. (**a**) Schematic representation of the initial morphologies of LPFO (below the critical micellar concentration) and TDMAO cylindrical micelles. (**b**) Time evolution of the SAXS intensity from a few milliseconds up to several seconds. (**c**) Modelling of selected SAXS profiles depicting the morphological change from the initially formed disk-like micelles to unilamellar vesicles when the size of the disks become larger. Adapted from J. Gummel et al. [62] ©the Royal Society of Chemistry 2011.

As shown earlier, SAXS has been extensively used for the characterization of multiscale structure in self-assembled systems. Elucidating the energetic pathways of self-assembly processes is not only of fundamental interest but also important for the rational design of many functional materials. In this respect, TR-SAXS experiments have provided valuable structural insights with model systems [17]. The stopped-flow rapid mixing that allows easy change of concentration or pH or ionic strength is a practical method for initiating the self-assembly process in the millisecond range. This approach has been used for probing the pathways of amphiphilic self-assembly including micellization, micelle–vesicle transition, micellar shape transformation, etc. [3]. In oppositely charged mixed surfactant systems that form unilamellar vesicles over a broad concentration range, the same final structure can be obtained by different routes involving disk-like or cylindrical and torus-like mixed micelles revealing the energetic stability of unilamellar vesicles [62]. Figure 7 illustrates a common pathway followed in the formation of unilamellar vesicles from oppositely charged surfactant micelles via disk-like mixed micelles. Such transient intermediate structures can be stabilized by admixing with an amphiphilic copolymer having an hydrophobic block length comparable to the surfactant bilayer thickness [63], providing long-term stability desired in their potential applications such as nanoreactors, nanocarriers, etc.

Similarly, the morphological transformations of surfactant micelles can be followed by stopped-flow TR-SAXS with millisecond range time-resolution. A nice illustration is the formation of long flexible cylindrical, worm-like, micelles when NaCl is added to an aqueous sodium dodecyl sulfate (SDS) solution [64]. The initial spherical micelles first transformed to elongated globular structures and then fused together to form long flexible cylinders approximately following a step-like polymerization type kinetics. In another investigation, the transition from spherical to cylindrical micelles upon mixing nonionic and anionic micelles revealed a two-step process involving unimer exchange between micelles followed by fusion of mixed spherical micelles to form cylindrical micelles [65]. In the case of a so-called platonic micellar system, a sharp transition from dodecamer to icosamer morphology was detected with the change in ionic strength [66]. Resolving this type of shape transformations require very precise measurements with SAXS intensities comparable on an absolute scale. TR-SAXS combined with stopped-flow mixing has also been applied in the investigation of phase transitions in amphiphilic liquid crystalline systems, which are relevant to therapeutic applications [67,68]. These systems are characterized by stimuli-responsive nanochannel architectures consisting of hydrophobic membraneous compartments and aqueous channels. When cationic lipid cubosome nanocarriers uptake neurotrophic plasmid DNA that loads into hydrated channels, lipoplexes with a multilamellar architecture are formed in millisecond timescale [67]. With the uptake of a neurotrophic protein, a sequence of transient structures has been observed where the lipid membrane curvature changed continuously resulting in a transition from inverted hexagonal–lamellar bilayer–bicontinuous cubic double diamond (Pn3m) to the final bicontinuous cubic gyroid (Ia3d) structure within a second [68]. In these studies, nanometer spatial resolution and millisecond time resolution make TR-SAXS unique and allowing to stitch together the limited real space information obtained from electron microscopy. This aspect of TR-SAXS has been key to the investigation of rapid structural changes during the osmotic shrinkage in a pharmacologically relevant liposome system [69]. Here, the quantitative analysis of the TR-SAXS intensity provided the time evolution of the radial electron density profile of the complex particles, which in turn revealed the structural dynamics of the liposomes at the nanoscale.

**Figure 8:**
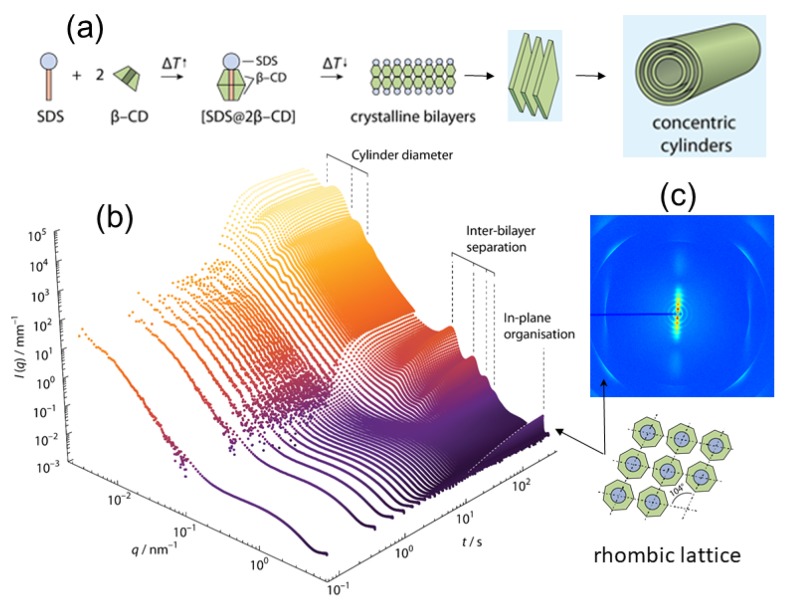
Hierarchical self-assembly of SDS and β-CD to form microtubules upon cooling a 10 wt% solution from 75 °C to 25 °C. (**a**) Schematic representation of the structural moieties involved in the assembly process. Two β-CD molecules and one SDS molecule form a capsid at higher temperature due to hydrophobic interactions as the interior of circular polysaccharides is slightly hydrophobic. Upon cooling, the capsids organize to crystalline bilayers, which then roll to form single-walled microtubes and subsequently the multilamellar structure develops by an inward growth. (**b**) Evolution of the SAXS and USAXS profiles showing the sequence of growth represented in panel (a). (**c**) 2D SAXS pattern from an oriented sample in the final state depicting the sawtooth Bragg peaks from a curved rhombic lattice, and form and structure factors of the multilamellar stacks within the tube walls. Adapted from J. Landman et al. [74] distributed under a Creative Commons Attribution Non Commercial License 4.0 (CC BY-NC).

The temperature and pressure are important parameters for controlling the nanostructure of lipid vesicular and liquid crystalline phases [70]. In stimuli-responsive drug-loaded formulations and vesicles loaded with gold nanoparticles, structural transformations and millisecond range intermediates have been detected by TR-SAXS combined with rapid temperature jump [70]. The lamellar–gyroid cubic phase transition in partially hydrated monolinolein was probed on the millisecond time scale following a pressure jump [71]. Results showed that the phase transition proceeds via a structural intermediate, the elastic energy in the bilayer drives the initially formed gyroid cubic phase to its equilibrium lattice parameter. Using highly swollen cubic phases of ternary lipid mixtures sensitive to temperature and pressure, it has become possible to achieve lattice dimensions comparable to those observed in biological systems [72]. Such systems offer the possibility for rational design of lyotropic phases suitable for investigating enzymatic studies, drug encapsulation, therapeutic delivery, etc. A spectacular example of thermally controlled hierarchical self-assembly has been revealed in a relatively simple system composed of a naturally abundant circular polysaccharide β-cyclodextrin (β-CD) and surfactant SDS (in 2:1 molar ratio) resulting in hierarchically organized microtubes of macroscopic dimension [41,73]. The self-assembly is induced by cooling the solution from 75 °C to 25 °C and SAXS revealed the in-plane ordering of the SDS-cyclodextrin capsids, the lamellar staking of the membranes and USAXS elucidated the structure of microtubes. TR-SAXS enabled following the exact sequence of steps in the assembly process of the membrane and their closing to form micron size microtubes, and uncover their subsequent inward growth as depicted in Figure 8 [74]. An important insight from the TR-SAXS experiment is that the multilamellar structure developed after the closure of the membrane to single-walled tubes by further nucleation and growth inwards which cannot be inferred from a static measurement. This type of self-assembly process may occur in a broader class of systems forming ordered multilamellar structures.

More recently, the assembly pathways in the formation of interpolyelectrolyte complexes (or polyelectrolyte coacervates) have been investigated by TR-SAXS combined with stopped-flow mixing [75,76,77,78]. For example, the complex formation between sodium polyacrylate (SPA) and polyallylamine hydrochloride (PAH) in aqueous NaCl solution was investigated by TR-USAXS for different NaCl concentrations from 0 to 1 M at equimolar concentrations of the monomer units [75]. Within the mixing dead time (∼2.5 ms), percolated aggregate-like structures were observed suggesting that the initially formed small charge neutral aggregates further assembled to form higher order agglomerates within a short time. The early stage time evolution of the molar mass of the large globular structure was found to be comparable with the Brownian-coagulation rate. The kinetics of complexation between the oppositely charged ionic/nonionic block copolymers with a branched star-shaped architecture and a thermoresponsive diblock (PNIPAM block) was investigated by mixing aqueous solutions (0.3 M NaCl) of both polymers for a charge ratio of 1 [76]. The complexation was essentially completed during the mixing and the resulting micelles remained stable over the measurement time, but their number density increased over the initial few seconds. Polyelectrolyte complex micelles formed from an anionic-neutral block copolymer, and a cationic-neutral block copolymer in aqueous NaCl solution exhibit different morphologies such as spheres or cylinders depending on their mixing ratios. A transition from sphere to cylinder and vice versa can be induced by mixing complex micelles with respectively pure cationic or anionic copolymers in aqueous NaCl solutions [77]. Morphological transformations in these systems take place on much longer time scale than in surfactant solutions. The cylindrical micelles transformed to spherical shape via the random scission of the cylinders along their contours in minute scale and the reverse process from spherical to cylindrical micelles was even slower with a high activation energy. The formation of polyelectrolyte coacervates with spherical core–shell morphology via a chain exchange mechanism has also been proposed [78].

### 3.3. Assembly of Biomacromolecular Complexes

**Figure 9:**
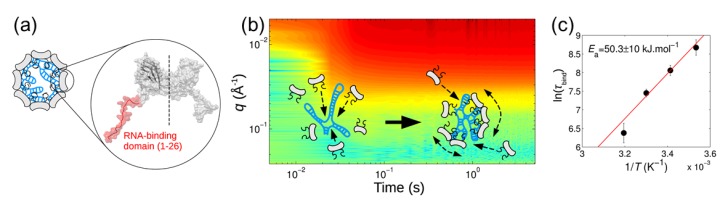
Assembly of icosahedral virus particles from the constituent capsids. (**a**) Schematic view of a virus particle with dimeric subunits and RNA genome. The expanded view displays the molecular structure of the subunit with one of its RNA binding domains. (**b**) Evolution of the SAXS intensity during the assembly process. The cartoon shows the rapid binding of subunits onto the genome and then the slow reorganization of the complex to the final structure. (**c**) Arrhenius plot of the binding time constant ($\tau_{bind}$) deduced from low *q* SAXS intensity at different temperatures, which provided an estimate of the activation energy ($E_{a}$). Adapted from M. Chevreuil et al. [84] licensed under the Creative Commons Attribution 4.0 International License.

Traditionally, TR-SAXS and related methods have been used for probing the structural dynamics in biomacromolecular systems which shed light on a broad range of biological functions. A well-known example is the study of muscle contraction along the pathway of physiological activation [79]. During this decade, TR-SAXS enabled probing in depth the self-assembly of virus particles [80,81], tubulin single rings [82], etc. from their constituting subunits. Detailed knowledge of virus assembly and disassembly under conditions similar to the host cells is important for the development of more effective vaccines. The rapid assembly of Simian vacuolating virus 40 (SV40) icosahedral particles from 12 pentameric viral protein capsids induced by a short RNA was investigated by stopped-flow mixing combined with SAXS. The observed kinetics was modelled by a two-state process without an intermediate [80]. The encapsidation process once nucleated at the RNA continued by a stepwise addition mechanism in which the growing nucleoprotein complex acts as an electrostatic antenna attracting other capsid subunits. In another study involving the self-assembly kinetics of norovirus capsid proteins, three species were found to contribute to the total SAXS intensities: dimers, intermediates comprising 11 dimers, and the icosahedral capsids made up of 90 dimers [81]. This biphasic kinetics involved a fast step in which dimers are assembled into intermediates, followed by a slow step in which intermediates interlock into capsids shedding new light on the generally accepted models for the assembly of norovirus capsids. The pH-driven disassembly of viral capsids derived from an icosahedral plant virus, the cowpea chlorotic mottle virus (CCMV), to dimers was found to be different from the assembly pathway and involved two distinct intermediates [83]. More recently, TR-SAXS was used to elucidate the nonequilibrium self-assembly dynamics of CCMV capsids packaging their RNA genome [84]. As shown in Figure 9, the experiment revealed the formation of amorphous complexes collectively with the genome acting as a template for the assembly, capturing a large number of subunits. These complexes relaxed into virions via a synchronous pathway in a slower process. The low *q* SAXS intensity was used to estimate the mean number of subunits bound on the genome as a function of time and the corresponding binding time constant, whereas the structural information was derived from the analysis of the scattering form factor. The temperature dependence of the relaxation time of the viral complexes allowed the determination of the activation energy of viral complexes to fully grown virus particles with the correct sequence of subunits. The binding energy of subunits on the genome was found to be moderate (≈7 $k_{B}T$) while the self-organization of nucleoprotein complexes into viruses involved a high energy barrier (≈20 $k_{B}T$). This barrier is significantly lower for a synthetic polyelectrolyte, such as poly(styrene sulfonic acid), as compared to RNA genome, but the resulting structure lacked the icosahedral symmetry.

In other TR-SAXS studies, the crystallization of wild type SV40 virus particles to body-centered cubic (bcc) structure upon dialysis with MgCl${}_{2}$ and their reentrant melting at higher MgCl${}_{2}$ concentrations were investigated [85]. Thermodynamic modeling of the transition at different salt concentrations suggested that the entropy of counterions is the driving mechanism. Swelling process of SV40 virus particles upon chelating calcium ions and reducing disulfide bonds was probed by SAXS and results provided a better insight into internal domain interactions and the binding of the capsid proteins in compact conformation [86]. TR-SAXS was also used for monitoring the large-scale conformational transitions of a two-state DNA origami switch from its open to closed conformation upon increasing the ionic strength in millisecond time scale, and found that the kinetics is close to the limit set by diffusion [87]. Time-resolved USAXS investigations of liquid–liquid phase separation kinetics in concentrated BSA solutions induced by trivalent salts and temperature revelaed spinodal decomposition and an arrested spinodal process [88]. Arrested and temporarily arrested states were observed in protein (bovine γ-globulin)–PEG mixtures upon quenching to the two-phase region depending on the magnitude of the temperature change [89]. In a more recent study, the influence of tuning the protein interactions on the spinodal decomposition process and formation of arrested states were systematically investigated [90].

### 3.4. In Situ Studies of Nucleation and Growth

Time-resolved simultaneous SAXS and WAXS methods have been extensively used for investigating the early stage of nucleation and growth in a variety of systems such as the pyrolytic synthesis of nanoparticles, precipitation of inorganic materials from supersaturated solutions [5], etc. This allowed the observation of nucleation process and clarifying the growth mechanism in these systems. More recent studies include in situ investigations of nucleation and growth of nanocrystalline particles such as quantum dots [91], porous metal organic frameworks (MOF) [92], etc. The different steps involved in the nucleation and growth of cadmium selinide (CdS) quantum dots from a surfactant mesophase precursor have been identified [91]. In this case, the surfactant lamellar phase transformed to micelles, and within which the activated monomers nucleated and formed nanocrystals stabilized by an outer surfactant layer. In the MOF case, the nanocrystal formation process involved the initial nucleation of amorphous clusters and their further growth by a coagulation mechanism and subsequent transformation to crystalline particles with specific zeolite topology, via intraparticle nucleation and structural reorganization [92]. In the case of strongly scattering samples, a stable free-jet of reactants can be combined with the SAXS set-up. This has allowed probing the prenucleation and nucleation stages in the sub-millisecond time as demonstrated in the case of CdS quantum dot formation [93].

**Figure 10:**
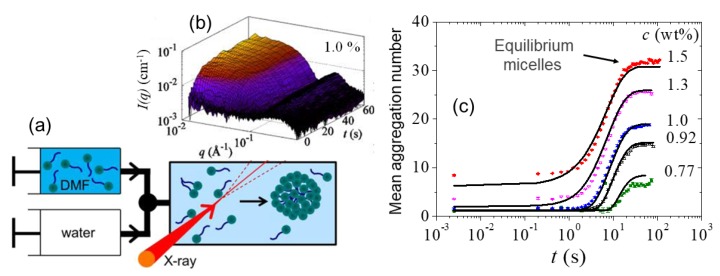
Nucleation and growth of surfactant (dodecyl maltoside) micelles from a solution of monomers in dimethylformamide (DMF) upon mixing with water. (**a**) Schematic drawing of the stopped-flow mixing device and the scattering cell with surfactants. (**b**) Time evolution of the SAXS intensity during the nucleation and growth process for 1 wt% solution. (**c**) Time evolution of the mean aggregation number derived from the modeling of SAXS intensities for different surfactant concentrations. The continuous lines show the good agreement with nucleation and growth model. Adapted from G.V. Jensen et al. [16] ©the American Chemical Society 2013.

TR-SAXS allowed a direct structural study of the micellization kinetics of surfactants that has been elusive for a century [16]. Figure 10 presents the experimental scheme and the main results which revealed a single step kinetics akin to nucleation and growth. The micelle formation is described as an insertion/expulsion process of unimers (isolated surfactant molecules) without any intermediate pre-micellar structures. A similar self-assembly and the opposite process of disintegration have been monitored in photosensitive surfactant (azo-benzene-based surfactants that can isomerize upon photon absorption) system by shining light of the appropriate wavelength (blue and ultraviolet, respectively) [94]. The kinetics appeared to follow a similar pathway of single step for micellization though the underlying kinetics is primarily determined by the quantum yield of light absorption. The demicellization involved a two-step process of the fast release of unimers followed by slower disintegration.

**Figure 11:**
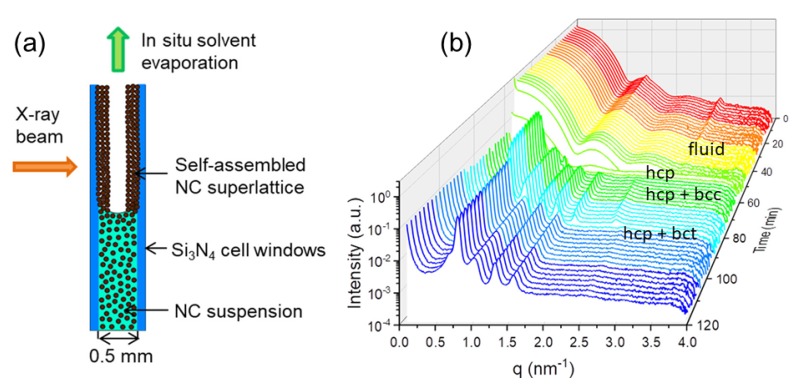
Evaporation-induced self-assembly of faceted PbS nanocrystals (NC). (**a**) Schematic view of the sample cell used for in situ measurements during controlled evaporation. (**b**) Time evolution of the SAXS profiles during the controlled solvent (heptane) evaporation from a 10 mg/mL colloidal suspension of nanocrystals. From the initial fluid-like colloidal solution, different crystalline morphologies, such as hcp, in coexistence with bcc and bct emerged as a function of evaporation time. The high angle peak in the fluid state is from the oleic acid grafting layer on nanocrystals. Adapted from I. Lokteva et al. [98] with permission from the American Chemical Society, ©ACS 2019.

A combination of SAXS and USAXS allowed the elucidation of the mechanism underlying the formation of ultrathin (∼2 nm) colloidal Cu${}_{2-x}$S nanosheets with well-defined shape and size [95]. The thermal decomposition of copper–dodecanethiolates usually leads to spheroidal Cu${}_{2-x}$S nanocrystals, but chloride stabilization of the stacks of lamellar copper-thiolate supramolecular complexes led to 2D-constrained stack-templated nucleation and growth, in which growth in the thickness direction is inhibited (allowing only the lateral growth). Another fascinating example is the formation of supraparticles (∼700 nm) from nanocrystals (∼12 nm) confined within oil droplets in an oil-in-water emulsion upon slow evaporation [96]. The nanoparticles consisted of an FeO core, a CoFe${}_{2}$O${}_{4}$ shell, and oleate capping ligands. Upon evaporation, the volume fraction of particles inside the oil droplets gradually increased up to ~20%, at which crystallization occurred instantaneously forming face-centered cubic (fcc) domains. Computer simulations showed that crystallization at such a low volume fraction is only possible if attractive interactions between colloidal nanoparticles are significant [96]. The evaporation induced self-assembly of lead sulfide (PbS) nanoparticles was studied by in situ SAXS [97]. The initial colloidal liquid ordered to a swollen hexagonal closed-packed (hcp) superstructure along the cell walls before transforming to bcc structure upon drying. Figure 11 illustrates the coexistence of different crystalline phases and a transformation to body-centered tetragonal (bct) structure upon complete drying [98]. The coexistence of two crystalline phases with a colloidal liquid has also been observed during the crystallization of a polydisperse colloidal system [99].

The crystallization of proteins is still not completely understood, and it is a bottleneck in crystallographic studies, especially for membrane proteins. SAXS studies have elucidated the structural signature of metastable intermediate phases and their role in the crystallization of model proteins, which is an important step towards understanding more complex proteins [100]. A two-step crystallization mechanism has been identified in globular protein solutions with multivalent counterions which undergo a metastable liquid–liquid phase separation prior to crystal growth [101]. SAXS revealed that proteins form clusters in the dilute phase which serve as the building blocks for nucleation, whereas the dense phase acts as a reservoir ensuring constant protein concentration in the dilute phase during crystal growth. In the presence of a divalent salt, an intermediate phase is formed as the first step, followed by the nucleation of crystals within this phase [101]. During the nucleation stage, the number of crystals increased with time, but crystal growth is slowed down due to the low mobility of proteins in the dense intermediate phase. In a next step, the intermediate phase is consumed by nucleation and slow growth, and, when it became sufficiently dilute, the number of crystals remained nearly constant, whereas crystals grew rapidly due to easy access to free proteins in the dilute phase.

Smart materials composed of adaptive hydrogels are macromolecules whose structure is very sensitive to external stimuli [102]. Chemical cross-links provide topological constraints and define their 3D morphology while their porous structure allows fast mass transfer, enabling very rapid structural adaption to changing environment. This structural evolution during the transformation of PNIPAM microgel having a flexible macromolecular network with a fuzzy interface to particle with well defined surface and homogeneous density was probed by TR-SAXS. Results revealed a two-stage kinetics involving a very fast process in which the collapsed clusters forming at the periphery (hollow core–shell) and a slower process in which the hollow core–shell structure transforming to a globular particle. This structural evolution appeared to be independent of the type of stimulus such as a temperature jump or a change of solvent quality suggesting the generality of the mechanism.

### 3.5. Equilibrium Dynamics

**Figure 12:**
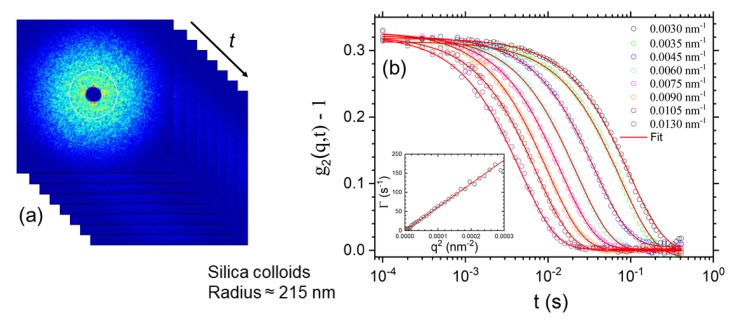
Multispeckle XPCS from a dilute colloidal suspension of silica particles (radius: ~215 nm) in water/3-methylpyridine mixture. (**a**) Representative 2D speckle patterns recorded in the USAXS range and the white circle indicating a given *q*. (**b**) Typical ensemble averaged intensity-intensity autocorrelation function ($g_{2}\left(q,t\right)$) and fits to an exponential decay with rate, Γ. The inset shows that $\Gamma =D_{0}q^{2}$, with $D_{0}$∼ 0.6 μm${}^{2}$ s${}^{-1}$, as expected for dilute Brownian particles.

The equilibrium microstructures of soft matter and many nanomaterials are not rigid and fluctuate in time due to ambient fluctuations, which could be of thermal origin or similar. There is an associated dynamics that can be Brownian motion in particulate systems, chain reptation in an entangled polymer melt, or membrane undulations in the case of a lyotropic system. It is often more challenging to probe this equilibrium dynamics as compared to the corresponding microstructure. DLS is a well-established method for investigating the dynamics in suspensions. However, there are limitations in terms of *q* range and concentrations which can be studied, especially when the sample becomes turbid. With the advent of third generation synchrotron sources, XPCS has emerged as an alternative method for probing the equilibrium dynamics in such systems [9,103,104,105]. The XPCS technique exploits the coherence properties of the X-ray beam and the measured scattering patterns display speckles as shown in Figure 12. The corresponding pattern registered with a partially coherent beam appears less grainy with nearly smooth intensity distribution since the speckle size in that case is much smaller than the detector resolution. The visibility or contrast of the speckle depends not only on the longitudinal and transverse coherence of the beam, but also on the angular resolution defined by the detector pixel elements. The $g_{2}\left(q,t\right)$ function of the fluctuating speckles reveal the dynamics within the system for a given *q* value as displayed in Figure 12. The time scale accessible by XPCS is determined by the scattering power of the sample, available coherent photon flux, and detector capabilities. The main applications of XPCS have been to probe the dynamics of colloids, especially those that are turbid in visible light [106] and slow dynamics in arrested systems such as gels [29] and glasses [27]. Faster dynamics at a given *q* can be accessed with a point detector [106]; however, an additional limitation is the onset of radiation damage with longer exposure of the sample to the X-ray beam. In dilute suspensions of Brownian particles, the dynamics is purely diffusive, and $g_{2}\left(q,t\right)$ decays by an exponential function and the decay rate is related to the diffusion coefficient, $D_{0}$.

The XPCS can be performed in both SAXS and WAXS configurations. The wide-angle XPCS provides access to atomic dynamics in relatively slow systems such as supercooled liquids and molecular glasses [104,105]. Recent studies of atomic dynamics include aging behavior of metallic glasses [107] and beam induced dynamics of oxide glasses [108]. For the investigation of arrested systems, XPCS performed with a 2D detector has a clear advantage, as multiple speckles along an azimuthal circle for any given *q* (multispeckle XPCS) are recorded simultaneously as demonstrated in Figure 12. This readily allows obtaining the ensemble averaged $g_{2}\left(q,t\right)$ of these non-ergodic systems. In addition, the two time correlation function, $g_{2}\left(q,t_{1},t_{2}\right)$, can be used to visualize the ageing behavior of the system as a function of time [3,105]. A common feature of these systems is hyperdiffusive dynamics characterized by a compressed exponential decay of $g_{2}\left(q,t\right)$ [9,105]. Although such a non-diffusive behavior could arise due to various reasons, a generally assumed mechanism is the presence of dynamic heterogeneities and associated internal stress relaxations [105,109].

**Figure 13:**
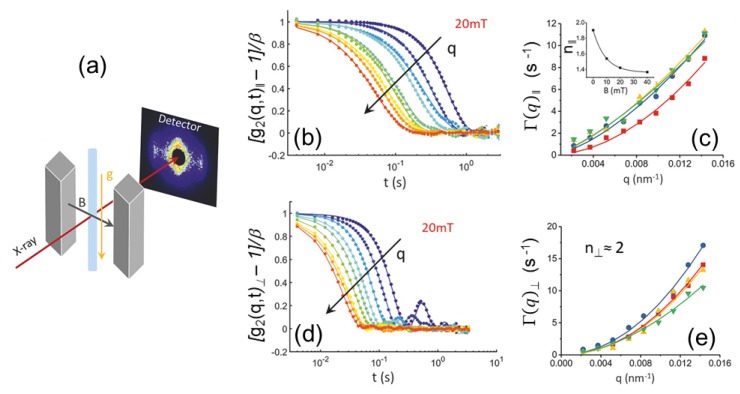
Anomalous dynamics of peanut-shaped anisotropic magnetic colloids (silica-coated hematite in an aqueous solution) in a magnetic field. (**a**) Schematic view of the experiment set-up used for multispeckle XPCS measurements. (**b**) Intensity autocorrelation functions ($\beta \;\equiv \;g_{0}$: ~0.3) along an horizontal sector parallel to the applied magnetic field (20 mT) and fits to compressed exponential functions (exponent about 1.5). (**c**) Anomalous dynamics showing $\Gamma {\left(q\right)}_{\|}\propto q^{n_{\|}}$. The inset displays the variation of $n_{\|}$ with magnetic field (B). (**d**) Corresponding intensity autocorrelation functions along the vertical sector perpendicular to the applied magnetic field. In this case, the dynamics is dominated by velocity fluctuations due to sedimentation [110]. (**e**) The Brownian part of the dynamics follows the expected behavior, $\Gamma {\left(q\right)}_{⊥}\propto q^{n_{⊥}}$, with $n_{⊥}∼$ 2. Adapted from A. Pal et al. [109] ©Weiley-VCH Verlag GmbH & Co. KGaA 2018.

The multispeckle XPCS is also a valuable tool for analysing the direction dependent dynamics, as illustrated in the case of shear flow [103] and sedimentation [110], where $g_{2}\left(q,t\right)$ involves both diffusive and advective contributions. Disentangling the information requires simultaneous measurements at many *q* values both along the vertical and horizontal directions. This enabled the measurement of velocity fluctuations at the early stage of sedimentation in brownian colloidal suspensions [110]. Similar approach is required for probing the anisotropic dynamics in an applied magnetic field [106,109] and in confined geometry of microfluidic channels [111]. In these cases, the measured microstructure and dynamics display strong anisotropy. Figure 13 illustrates the observed anomalous dynamics and chain formation by peanut-shaped anisotropic magnetic particles in a magnetic field. Fast multispeckle XPCS has allowed the investigation of phoretic dynamics of colloids [112] and active motions of Janus colloids [113] in phase separating solvent mixtures. The availability of fast 2D detectors such as the Eiger-500k pixel detector has enabled multispeckle XPCS measurements in the sub-millisecond range [114].

Another important application of XPCS is to probe dynamics at surfaces and interfaces [9] and in films [115] using grazing incidence geometry (GIXPCS). For instance, this method has enabled quantitative studies of capillary wave fluctuations on liquid surfaces and in polymer films [9] and to verify the predictions of hydrodynamic continuum theories. The heterogeneous dynamics of 2D gels was probed in Langmuir films [116], which allowed for the derivation of the fourth order time correlation function and to detect the characteristic time of dynamical heterogeneity. For off-specular geometries, nanoparticles were used as tracer particles to probe the interior dynamics in polymer films and monolayers [9]. An induced dynamical arrest transition was observed in phospholipid/nanoparticle monolayers that is again featured by an hyperdiffusive dynamics [115].

The scope of XPCS will be significantly enlarged with the availability of extremely brilliant synchrotron sources and even faster pixel array detectors. The feasibility of studying ultrafast dynamics using an alternative method—speckle visibility spectroscopy—has already been demonstrated at the X-ray free-electron laser (XFEL) [117]. However, this method has limitations when studying complex relaxation processes involving non-exponential $g_{2}\left(q,t\right)$ and non-monotonic *q* dependence. As a result, the multispeckle XPCS at synchrotrons remains a more straightforward tool for investigating complex dynamics involving multiple length and time scales.

### 3.6. Flow-Induced Structures

**Figure 14:**
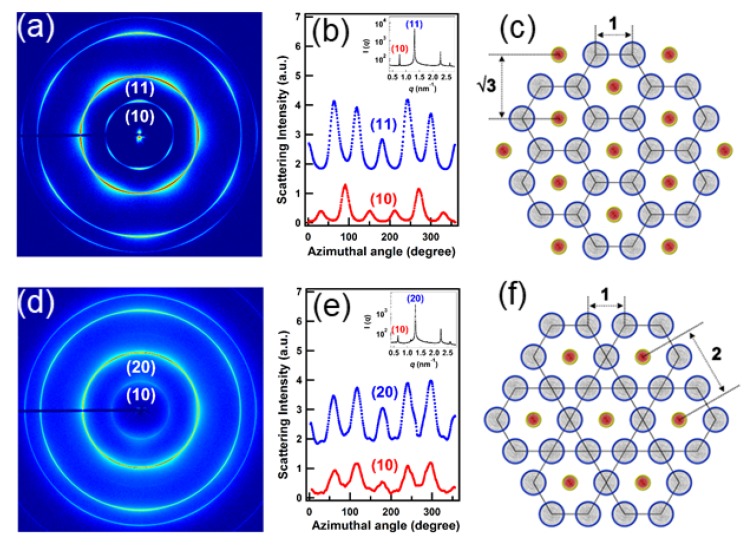
Shear-induced alignment of binary supperlattices formed by hexagonally packed cylindrical micelles of nonionic surfactants penta(ethylene glycol) monododecyl ether ($C_{12}E_{5}$) in water and rod-like micelles of polymerizable cationic surfactants, n-alkyltrimethylammonium 4-vinylbenzoate ($C_{n}$TVB, with alkyl chain length, n = 10, 12, 14 or 16. (**a**) Oriented SAXS pattern along the flow direction for $p-C_{14}TVB/C_{12}E_{5}$/water (10/45/55 weight ratio). (**b**) Azimuthal variation of intensities along the (10) and (11) reflections (which make the peak position ratio of 1:√3) for $AB_{2}$ type structure. Inset shows the azimuthally averaged intensity. (**c**) Schematic representation of a $AB_{2}$ type binary superlattice with an hexagonal array of $p-C_{n}TVB$ rods (smaller spots) intercalated in an honeycomb lattice of $C_{12}E_{5}$ cylinders. (**d**) Oriented SAXS pattern along the flow direction for $p-C_{10}TVB/C_{12}E_{5}$/water (5/45/55 weight ratio). (**e**) Azimuthal distribution of intensities along the (10) and (20) reflections (which make the peak position ratio of 1:2) for $AB_{3}$ type structure. Inset displays the azimuthally averaged intensity. (**f**) Schematic representation of a $AB_{3}$ type binary superlattice in which an hexagonal lattice of $p-C_{10}TVBs$ is embedded in a kagome lattice of $C_{12}E_{5}$ cylinders (larger spots). Adapted from S.-H. Lim et al. [122] licensed under the Creative Commons Attribution 4.0 International License.

The characteristic feature of soft matter systems is that they are easily deformable by an applied shear stress. As a result, a large number of investigations have been performed to elucidate the microstructure as a function of applied shear rate or shear stress [3,118]. A major goal, which is also of great practical interest, has been relating the microstructure to rheological properties. The high degree of collimation and small size of synchrotron X-ray beams are important for probing highly ordered systems. For example, elucidating the order–disorder transition underlying shear thickening in dense suspensions of colloidal particles subjected to different oscillatory shear stresses [119]. When combined with large amplitude oscillatory shear, XPCS can detect nonlinear rheological behavior such as yielding and plastic flow during an oscillation [103]. For example, the local signature of yielding during large amplitude oscillatory shear has been detected in concentrated oil-in-water nanoemulsions [120]. The shear flow is also an elegant method to orient the sample and deduce high resolution structural information as shown in the case of hexagonal honeycomb and kagome superlattices in surfactant lyotropic hexagonal phase intercalated with single-walled carbon nanotubes [121] and polymer nanorods [122], respectively. Figure 14 illustrates the shear-induced orientation of binary supperlattices with hexagonal honeycomb and kagome structures.

Model colloidal fluids in nanofluidic channel arrays displayed strongly anisotropic structure factor due to the confinement [111]. This confirmed the theoretical prediction of a confinement-induced anisotropy of the pair correlation function of a fluid. SAXS allowed the characterization of microstructure within the concentration polarization layer during the cross-flow ultrafiltration process of cellulose nanocrystal dispersions, including the orientation and packing as a function of the distance from the membrane, and link to the concentration polarization and identifying the fouling layer near the membrane [123].

Recent advances in microfabrication techniques have enabled the realization of microfluidic devices that can deliver small quantities of liquids (nanolitres) and combine with X-ray scattering experiments [124]. They also offer new possibilities for the investigation of soft matter in low Reynolds number flows and under confinement [125]. When combined with microbeam X-ray scattering measurements, the local structure in the flow field can be mapped, thereby offering the possibility of investigating unexplored nonequilibrium states of soft matter. A straightforward application is the investigation of flow alignment of complex fluids in microfluidic channels especially from the point of view of materials processing [125]. For example, the orientation dynamics of a nematic director in a thermotropic liquid crystal was probed under different flow rates and boundary conditions [125]. Microfluidics are convenient environment for probing conformational changes of proteins and other biomacromolecules upon change of the buffer condition [124], and nucleation and growth of a specific polymorph that may be difficult to isolate in the bulk as shown in the case of calcium carbonate precipitation [126]. Another important advantage is that the radiation damage can be minimized by the continuous flow without consuming too much sample but at the expense of a reduced scattering signal to background ratio. A microfluidic environment can be used to investigate biological supramolecular assemblies by mimicking conditions involved in blood vessels and explore the new physics of such systems under confinement [124].

**Figure 15:**
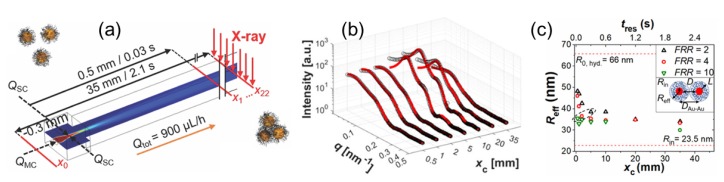
Probing the hydrophobic collapse of grafted polystyrene chains on gold nanoparticles in THF upon mixing with water. Initially, repulsive particles became attractive and clustered upon collapse of the grafted chains. (**a**) Schematic representation of the 3D flow-focusing microfluidic reactor with a middle channel (MC) and two side channels (SC), and the simulated concentration pattern of THF/water mixture. The X-ray beam passed orthogonal to the flow and the lateral focusing directions. (**b**) Evolution of the SAXS profiles of the particles as a function of the distance from the mixing point ($X_{C}$) for a flow rate ratio (FRR) of 2. The peak corresponds to the structure factor of the particles upon clustering and modeling of which provided an effective size of particles. (**c**) The variation of effective particle radius as a function of $X_{C}$, which is equivalent to a residence time ($t_{res}$) given by the flow rate, for different FRR. The chain collapse transition occurred in the millisecond time scale. Adapted from S. Merkens et al. [127] with permission from the American Chemical Society, ©ACS 2019.

Using SAXS combined with a microfluidic cell and optical spectroscopy techniques, the hydrophobic collapse of grafted polystyrene chains on gold nanoparticles suspended in tetrahydrofuran (THF) was observed upon mixing with a poor solvent (water), as depicted in Figure 15 [127]. The thickness of polymer shell derived from the SAXS analysis and numerical simulation of the solvent composition allowed the mapping of the interaction energy between particles, and it was found that the rate of hydrophobic collapse depends on water concentration, ranging between 100 and 500 nm/s, and that the polymer shell collapses prior to the onset of clustering of gold particles.

### 3.7. High Resolution Diffraction and Imaging

This subsection describes some of the studies which will greatly benefit from the upgrades of synchrotrons such as the ESRF extremely brilliant source (EBS) or the Advanced Photon Source upgrade (APS-U). Indeed, time-resolved SAXS and GISAXS and USAXS will gain from the high brilliance in terms of time and angular resolutions, whereas XPCS takes advantage of the increase in transverse coherence in dynamic studies. The order of magnitude increase in angular resolution will be even more significant for small-angle X-ray diffraction (SAXD). Similarly, the enlarged transverse coherence could improve the spatial resolution in coherent diffractive imaging (CDI) down to 10 nm range. The CDI is a lensless imaging method based on phase retrieval by an iterative procedure from an oversampled (as compared to the Nyquist period) speckle pattern from a noncrystalline specimen [10]. Together with tomographic reconstruction, this method can yield 3D images of micron sized objects.

Colloidal crystals are another class of systems which require very high angular resolution in order to elucidate the long-range order and defects within them [8]. Very high-resolution SAXS (a few microradian) has been pivotal in distinguishing different crystalline as well as liquid crystalline phases formed by anisotropic colloidal particles [8]. This has been nicely illustrated in the case of superball (shape between a sphere and a cube) particles, which showed a plastic crystal phase with translational order and orientational disorder and two distinct rhombohedral crystalline phases with different stacking variants [128]. Transition between these solid phases occurs depending on the osmotic pressure. The large transverse coherence of the X-ray beam is essential for revealing the long range order that may be several orders of magnitude larger than the typical 2π/q scale probed by the measurement [8]. Elucidating the different liquid crystalline phases coexisting in suspensions of clay particles with large aspect ratios (diameter: approximately 100 nm to 1 μm thickness: ~1 nm) is a challenge due large periodicity and small domain sizes [129], e.g., differentiating the lamellar phase from a nematic phase with strong stacking local order, the so-called columnar nematic. The high-resolution SAXS measurements allowed the clear identification of such phases in dilute aqueous suspensions of synthetic Sb${}_{3}$P${}_{2}$O${}_{14}$ nanosheets. Analysis of their sharp X-ray reflection profiles in the direction perpendicular to the director revealed that two coexisting mesophases are a columnar nematic and a lamellar phase with domain sizes of about 20 μm (deduced from the width of the Bragg peak), which corresponds to about 600 nanosheets.

An important motivation for studying long-range ordered colloidal systems has been from the point of view of potential photonic materials [8]. More recent investigations employed high-resolution SAXS for elucidating the structural basis of colors in biophotonic specimens [57,130,131]. High-resolution studies of butterfly single scales revealed the 3D photonic nanostructure as a single network of gyroid morphology formed by chitin and air which is reminiscent of cubic phases observed in amphiphilic soft matter systems such as surfactants, block copolymers and lipids in water or block selective solvents. Results supported the hypothesis that color-producing protein and air nanostructures in feather barbs are self-assembled by arrested phase separation of polymerizing β-keratin [56], and the nanostructure can be varied continuously by regulating the time the keratin network is allowed to phase separate before mobility in the system is arrested [131]. In arthropod scales and setae, a richer nanostructural diversity, including triply periodic bicontinuous networks, close-packed spheres, inverse columnar, perforated lamellar, and disordered spongelike morphologies have been reported [130]. The challenge in investigating these systems is that not only high angular resolution required to resolve long periodicities, but also the beam cross section should be small to probe the gradient in color (e.g., feather barbs).

SAXD continued to be valuable for structure–function studies in biological tissues and fibers. A notable example is muscle, where SAXD allows probing the different regulatory states during the contraction and relaxation [79,132]. For example, high-resolution SAXD together with sarcomere-level mechanics enabled the identification of a downstream mechanism in muscle regulation, namely the thick filament (myosin) based regulation, which involves a mechano-sensing role of the myosin filament [133]. Most myosin motors are in a constitutively off state and the transition to on state occurs based on the load sensed by the myosin filament. This additional mechanism preserves the high metabolic efficiency and offers a molecular basis for the length-dependent activation in cardiac muscle [134]. The approach developed for simultaneous investigation of micrometer scale supramolecular organization and nanometer scale filamental protein movements is useful for identifying the elements of muscle dysfunction [79]. Another example is the investigation of the ultrastructural mechanics of collageneous tissues. By measuring the time-dependent changes in fibrillar structure during in situ tensile testing, the ultrastructural mechanics could be probed at different chemically induced mechanical states [135]. The variable interfibrillar stiffness can be related to the mechanism of mutability at the nanoscale. A model of stiffness modulation via enhanced fibrillar recruitment explains the underlying biophysical mechanism which may have applications in development of new types of mechanically tunable biomaterials.

**Figure 16:**
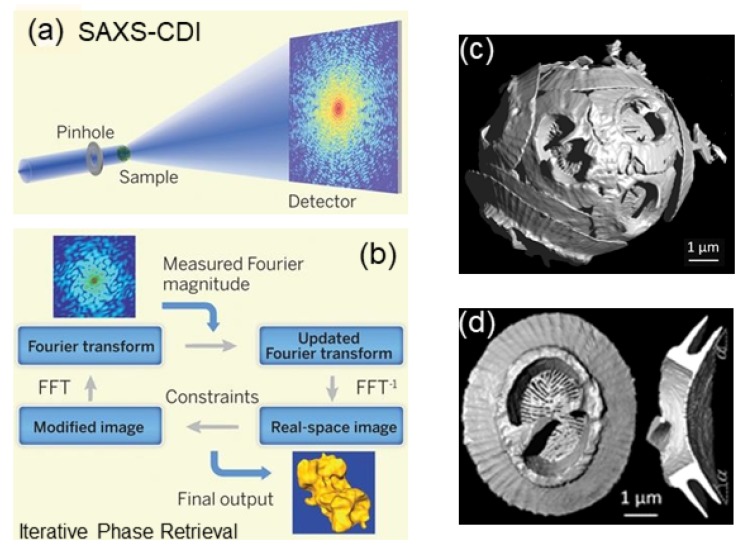
(**a**) The optical and detection schemes used in SAXS-CDI. A plane wave illuminates the noncrystalline specimen, and an oversampled speckle pattern is recorded by a high-resolution detector. (**b**) The phase retrieval procedure involves iterating back and forth between real and reciprocal space. In each iteration, various real space physical constraints such as positive electron density, partially overlapping regions, etc. are imposed, while the measured Fourier amplitude is updated in reciprocal space. Panels (a,b) are adapted from J. Miao et al. [10] with permission from the American Association for the Advancement of Science, ©AAAS 2015. (**c**) 3D images of coccospheres of *G. oceanica RCC1314* reconstructed by tomographic SAXS-CDI. (**d**) Distal view of the coccolith and a side view along the major axis after sectioning half of the extracted coccolith. Panels (c,d) are adapted from T. Beuvier et al. [142] licensed under the Creative Commons Attribution 4.0 International License.

CDI is an emerging method suitable for obtaining real space images of biological specimen and nanomaterial assemblies in 3D. Figure 16 illustrates the optical and detection schemes employed for CDI in the SAXS configuration (SAXS-CDI) and the iterative phase retrieval procedure [10]. The resolution is primarily limited by the radiation damage and the *q* range of the speckle pattern with sufficient signal. Radiation sensitive biological cells are typically imaged in the frozen hydrated state [136,137]. Resolution obtained are in the range of 75 to 100 nm with *Neospora caninum* [136] and malaria-infected human erythrocyte [137] cells, which can barely resolve the organelles within. At present, the best 3D resolution obtained is in the range 30 to 50 nm for strongly scattering samples such as porous calcium carbonate [138] or silica [139] microparticles, titania sponge [140], or gold nanoparticles in polymeric capsules [141]. A very clear illustration of the 3D imaging capability by CDI is the recent study of coccolithophores, unicellular algae of size a few microns, that achieved a resolution close to 30 nm [142]. The images displayed in Figure 16 reveal coccoliths morphology in unprecedented detail and quantitative analysis established a relationship between their mass and the number of calcite segments [142]. In addition, a correlation between their overall dimension and size of certain organelles within the cell was noticed which may correspond to different stages of their growth.

With the increase in brightness and coherence, and advances of detector technology with better spatial resolution and larger area, the 3D imaging resolution is expected go down to 10 nm or so. An important goal is to enable imaging of functional systems in nanoscience and biology with nm range resolution. In this respect, the potential of CDI for imaging a transient process has been demonstrated [143]. Indeed, the radiation damage is a limiting factor as the imaging requires at least an order of magnitude higher radiation dose than the corresponding diffraction measurement. Therefore, SAXD will remain relevant because the nanoscale structural and dynamical information, albeit in reciprocal space, can be derived prior to the onset of radiation damage. When investigating complex functional systems by CDI, simultaneous analysis of SAXD could provide additional constraints to the image reconstruction procedure.

## 4. Surface and Interface Studies

This section describes some representative investigations of nanomaterials and soft matter at surfaces and interfaces using X-ray scattering methods. X-ray and neutron scattering can derive unique information at the nanoscale which is not easily obtained by other surface sensitive techniques [3,144].

### 4.1. Nanoparticles at Interfaces

**Figure 17:**
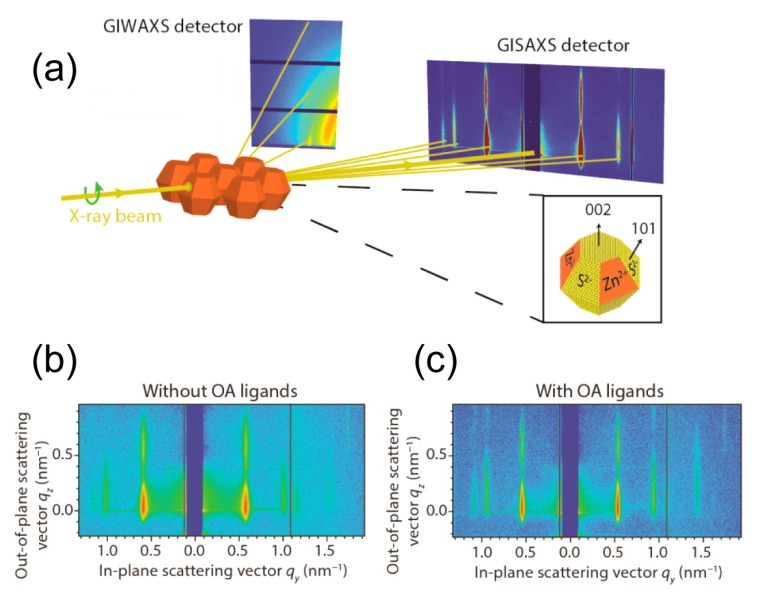
(**a**) Schematic representation of the in situ simultaneous GISAXS and GIWAXS measurements of 2D zinc sulphide (ZnS) nanocrystal superlattice structure developing at a liquid–vapor interface. The inset shows an enlarged wurtzite hexagonal bifrustum-shaped ZnS nanocrystal. Bottom panels show GISAXS patterns during the formation of the 2D hexagonal ZnS superstructure with scattering rods in the in-plane scattering direction (**b**) without and (**c**) with oleic acid (OA) ligands which induce atomic scale alignment of nanocrystals and promote the superlattice formation. Adapted from Van der Stam et al. [148] with permission from the American Chemical Society under ACS Author Choice License.

**Figure 18:**
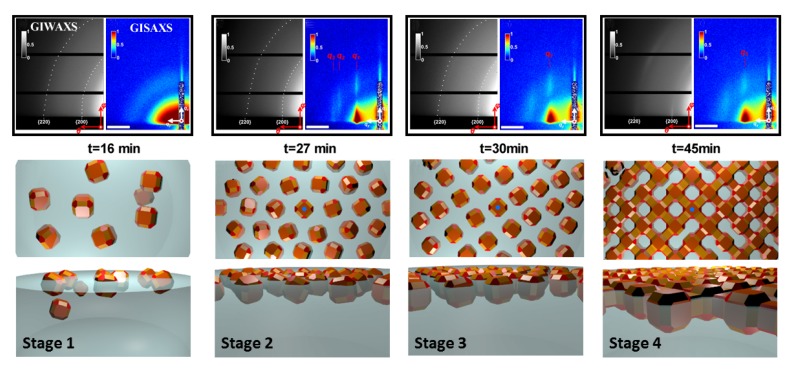
GISAXS and GIWAXS measurements during the self-assembly of PbSe nanocrystals. The lower panels display the corresponding structural models as side view (middle row) and top view (bottom row). All GISAXS scale bars denote 1 nm${}^{-1}$. Adapted from Geuchies et al. [149] ©Springer Nature 2016.

Two-dimensional self-assembly of colloidal nanocrystals and nanoparticles at liquid–liquid interfaces is of both fundamental scientific interest and vast technological relevance. For example, these structures are promising candidates for the fabrication of new metamaterials, photovoltaic devices, miniature light sources, magnetic storage media, etc. XRR enables measuring the location of nanoparticles with respect to the interface of immiscible liquids and determine quantities such as contact angle, binding energy, and interparticle distance, which control their lateral organization [145,146]. A GISAXS study of the CdSe-CdS core–shell nanorods [147] self-organization at the liquid–vapor interface into 2D crystals revealed an unexpected change from parallel to perpendicular orientation of the rods with respect to the liquid surface. Simultaneous measurements of GISAXS and GIWAXS during evaporation-driven precipitation of wurtzite ZnS bifrustum-shaped nanocrystals at toluene–air interface [148] provided, for the first time, information about different stages of development of 2D superstructure with long-range orientational order from a disordered assembly of nanoparticles as depicted in Figure 17. Here, the atomic plane orientation was induced by oleic acid ligands, thereby promoting the superlattice formation. Further application of simultaneous GISAXS and GIWAXS measurements to probe the self-assembly of PbSe nanocrystals during evaporation at liquid–vapor interface [149] demonstrated that particles with a different shape, e.g., truncated cube, also form atomically coherent 2DM. This behavior of nanocrystals with active facets appears to be general as suggested by these multiple observations. The experiment revealed for the first time the sequence of four phase transitions during the self-assembly process of nanocrystals on a liquid interface as shown in Figure 18. In stage 1 of the self-assembly process, the apolar solvent of the suspension gradually evaporates and the increasing concentration forces nanoparticles to slowly attach to the interface. During stage 2, most of the apolar solvent has evaporated, and the nanocrystals self-assemble into an energetically favored dense hexagonal array. In stage 3, this hexagonal monolayer starts to deform towards the final square lattice. In stage 4, the nanoparticles are in close proximity and they atomically fuse together to form crystalline bridges between adjacent planes. The insights gained from these observations may lead to bottom-up routes for fabrication of diverse 2D electronic or photonic materials based on nanocrystals. One of the important ingredients for the self-assembly of nanoparticles at the liquid interface is surfactants or ligands on their surface. Final structure depends on the length of the grafted moelcules, type and exchange between nanoparticles, and the bulk liquid that plays the role of a draining reservoir. Time-resolved GISAXS study of PbS nanocrystal self-assembly on the acetonitrile–air interface shed light on the role of oleic acid exchange by tetrabutylammonium tetrathiafulvalenedicarboxylate molecules on the final structure of the assembly [150]. It was found that the final interparticle spacing is precisely one molecular length of the bidentate molecule, suggesting that it rigidly connects adjacent nanocrystals. It is remarkable that nanoparticles assembled at the liquid surface allowed subsequent modifications without deterioration of their structural integrity. This work is intended to help improving the long-range order in nanocrystal superlattices and to provide guidelines for optimized ligand exchange conditions.

Ordering of the functionalized monodomain magnetic nanoparticles assembled at the liquid–vapor interface depends on the combined van der Waals and magnetic interactions. There is an optimal nanoparticle size, in other words, magnetic moment, to achieve a large area of a highly ordered monolayer [151,152]. Binary mixtures of nearly monodisperse iron oxide particles of 10, 15, and 20 nm in diameter self-assemble differently when spread on a liquid surface. XRR and GID revealed that a 3:1 binary mixture of 10 nm and 20 nm particles self-assemble to a structure where the pristine monolayer of 10 nm particles is perturbed by the larger particles. Nontrivial mixing causes an enlargement of interparticle distance but maintains the symmetry of a 2D lattice of smaller nanoparticles [153]. Nanoparticle layer formed at liquid–vapor interface can be transferred on to a solid substrate via Langmuir-Blodgett (LB) method to produce a mono or multilayers. XRR study of structural aging of a freshly prepared LB multilayer of dodecanethiol-encapsulated Au nanoparticles on a year scale revealed the gradual transition from periodic alignment of nanoparticles along surface normal to a disordered state with decrease of film thickness and coalescence of Au core [154].

Contrary to the Langmuir monolayer of insoluble surfactant coated nanoparticles, a Gibbs monolayer formed from soluble particles allows additional degrees of freedom to arrange 2D patterns at a fluid interface, e.g., hydrophilic SiO${}_{2}$ particles in a solution of cationic surfactant, cetyltrimethylammonium bromide (CTAB), form complexes and move to the oil–water interface, organizing in 2D arrays with interparticle distance varying over a large range depending on CTAB concentration and the ionic strength [155]. Lattice parameters of the 2D structure obtained from GISAXS are in good agreement with atomic force microscope (AFM) measurements at the liquid–liquid interface.

Many organic molecules tend to form nanoclusters, which in turn order into superstructures. Surfactants with perfluorocarbon blocks in their chains spread on water surface spontaneously forming nearly monodisperse hemi-micelles. The size of these micelles can be controlled by the molecular length and the block ratio. These molecules are used to design new types of colloidal systems, targeting potential medical applications. The structure and lateral correlation of fluorocarbon-hydrocarbon tetrablock di($F_{10}H_{m}$) domains at the air–water interface [156] have been determined by quantitative analysis of GISAXS data. The lateral correlation was found to extend more than 14 times the distance between the nearest neighbors. Simulation of the 2D GISAXS intensity in terms of the form and structure factors suggests that di($F_{10}H_{m}$) domains take a hemiellipsoid shape. Both major and minor axes of the hemiellipsoids monotonically increased in response to the elongation of the hydrocarbon blocks, which can be explained by the concominant increase in van der Waals interaction. Studies on the $F_{n}H_{m}$ familiy of fluorocarbon-hydrocarbon, namely $F_{10}H_{16}$, showed that the inter-domain correlation can reach a distance that is more than 25 times larger than the size of individual domains [157]. This opens the possibility towards the hierarchical design of mesoscale domains of self-assembled small organic molecules to large 2D structures. GISAXS measurements on hemi-micelles with an aspect ratio (diameter/height) necessitated a very long detector in $q_{z}$ direction as the Bragg rods spacing is very short in comparison with the extension of rods. An intriguing question about this system is how the hydrogenated and fluorinated moieties are arranged with respect to the water surface before and after the collapse of the film. An in situ GISAXS study of multilayer films of semifluorinated alkanes (SFA) $F_{8}H_{18}$ at the air–water interface provided evidence that the first layer in contact with the water subphase, buried below the overlayers, exhibits the same supramolecular hexagonal structure that is observed in the monolayer before the collapse, at non-zero surface pressure [158]. This result demonstrates the major role of the interactions between the first layer of SFA and the water subphase in the formation of the structure. Disk-like hemi-micelles of SFA on the water surface stay nearly monodisperse and do not coalesce upon increasing surface pressure. To understand this behavior, a comprehensive analysis of Langmuir monolayer of $F_{8}H_{18}$ SFA by in situ GISAXS was carried out. Results showed that the structure of their condensed phase consists of domains of upright molecules surrounded by molecules which are lying down [159]. Such a model explains the non-coalescence of the domains and structuration is driven by the interaction of the lying molecular dipoles with the dipole of water at the surface.

In view of rapid progress in the field of spintronics, multilayer nanostructures consisting of alternating ferromagnetic and semiconductor layers have received significant attention. Amorphous multilayer nanostructures are of particular interest since they combine high magnetic and magnetoresistance characteristics with better structural stability due to the absence of grain boundaries and the homogeneity of interfaces. GISXAS and neutron reflectivity structural studies of [(Co${}_{45}$Fe${}_{45}$Zr${}_{10}$)${}_{x}$(Al${}_{2}$O${}_{3}$)${}_{100-x}$/a-Si:H]${}_{m}$ (*m* = 36) nanocomposite as a function of the a-Si:H layer thickness established that the magnetization and electrical resistance of the film is a nonmonotonic function of the a-Si:H layer thickness [160]. Both characteristics were found to be at a minimum for a structure with a semiconductor layer thickness of 0.4 nm, which is identified to a weak ordering of Co${}_{45}$Fe${}_{45}$Zr${}_{10}$ grains. A GISAXS study on multilayer of nanocomposite [(Co${}_{40}$Fe${}_{40}$B${}_{20}$)${}_{34}$(SiO${}_{2}$)${}_{66}$]/[C]${}_{47}$ did not reveal an ordering of (Co${}_{40}$Fe${}_{40}$B${}_{20})$ nanoclusters in the multilayer along the surface normal; however, a weak lateral organization was found [161]. The homogeneous semiconductor interlayer identified there leads to modification of the metal–insulator transition that drives the changes in the magnetic and electrical properties.

Mesoscale structures and processes are among the most intensely studied topics in physics, chemistry, and nanotechnology. Being intermediate between the atoms or molecules and macroscopic or continuum length scales, they often require multiscale theories and experimental approaches for gaining a deeper understanding. Although the GISAXS is a very powerful technique to study mesoscale structures, the information from scattering patterns is not often fully exploited. Only geometrical information such as peak positions and widths are usually extracted. Unravelling the structural properties of mesostructured thin films containing highly organized internal 3D structures remain a challenging issue because of the lack of efficient algorithms, which allow prediction of the GISAXS intensity patterns. A quantitative GISAXS analysis using the distorted wave Born approximation (DWBA) was performed in the case of CTAB-templated mesoporous silica thin films [162]. Tiny features in the GISAXS intensities of these films produced by evaporation-induced self-assembly showed that nanopores are not spherical but slightly deformed, elliptical, along the surface normal. Grazing incidence scattering under certain conditions provides information similar to XRR but enables time-dependent structural studies of rapidly evolving interfaces with poorly ordered 2D structures. An example is a study of protein layer under lateral compression that enabled measurement of film elastic properties [163,164].

### 4.2. Model Biological Membranes

Proteins and lipids are often used in biomimetic systems and biomedical applications in which the role of active components is probed. Langmuir layers at the air–water interface continue to serve as a simplified model for biologically relevant membranes. Although the Langmuir layer is far from the real bilayer in a living cell membrane, this approach allows easy control of thermodynamic parameters of the membrane (surface pressure, temperature, and composition), mimic the inner or outer membrane environment and follow structural modifications upon adsorption of proteins, peptides, and macromolecules from the aqueous sub-phase with selected pH or ionic strength. For example, XRR measurements on a DPPC Langmuir monolayer interacting with hyaluronan of different molecular weights and ions dissolved in water [165] yielded a better understanding of the joint lubrication role. The combination of XRR and GIXF permitted studying the adsorption of histidine-tagged cadherin proteins on to lipid monolayers at the air–water interface [166], and the ion distribution at the hydrophilic surface of peptide monolayers [167] and the impact of lipid oxidation on the layer structure [168].

High-energy X-rays permit an extension of the conventional Langmuir techniques to study bulk liquid–liquid interfaces and therefore biologically relevant structures formed at these interfaces. For example, an investigation of the alternating electric-field effect on supported lipid membranes by GISAXS revealed a strong decrease in the membrane tension (up to 1 mN/m) and a large increase in membrane rigidity (up to 300 $k_{B}T$) for local electric potentials of the order of 1 V [169]. These results were quantitatively interpreted in terms of the amplification of membrane surface charge fluctuations resulting in lower tension and increased interaction between charges in the electric double layer leading to the increase of bending rigidity [169]. These effects eventually destabilized the lipid bilayers and transformed to unilamellar vesicles. In many cases, membranes are formed on a polymer cushion in order to shield the lipids and proteins from detrimental interactions with the solid support. Adsorption of a model cell membrane on to hydrated biopolymers located at the solid–liquid interface (the cushion layer mentioned above) allowed adjusting the interfacial interactions and creating more biologically relevant membranes [170]. High energy XRR is mandatory for studies of buried interfaces, yielding the equilibrium membrane–substrate distances, which can quantitatively be modeled by computing the interplay of van der Waals interaction, hydration repulsion, and repulsion caused by the thermal undulations of the membrane.

**Figure 19:**
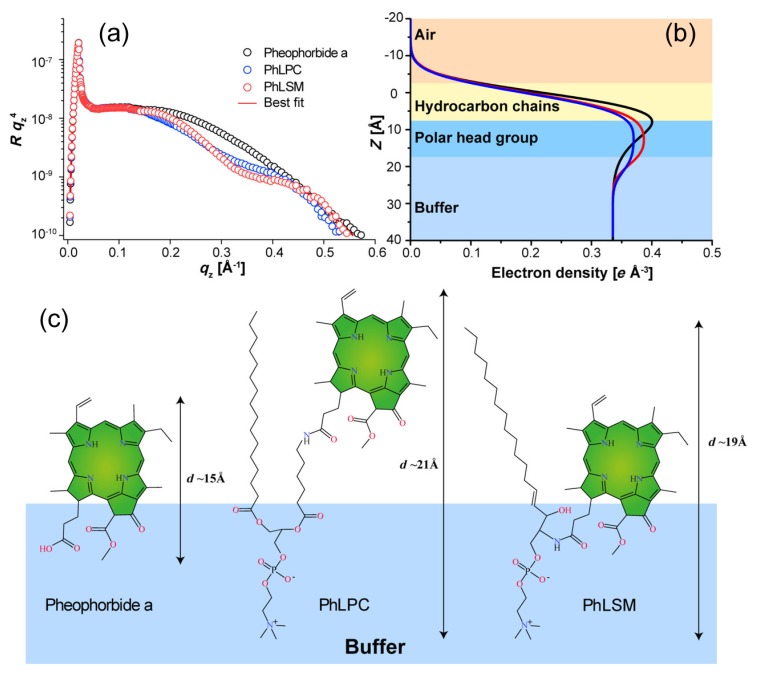
(**a**) XRR curves of monolayers of a Pheo-a derivatives at a surface pressure of 30 mN/m. The solid lines represent the best fit models to the experimental data. (**b**) The reconstructed electron density profiles along the z-axis. (**c**) Schematic representation of the orientation of Pheo-a derivatives at the air-buffer interface. Adapted from Massiot et al. [171] with permission from the John Wiley and Sons ©Wiley 2018.

Lipid-porphyrin conjugates are considered, nowadays, as promising building blocks for the conception of supramolecular structures with multifunctional properties, required for efficient cancer treatment by photodynamic therapy. Two lipid-porphyrin conjugates synthesized by coupling pheophorbide-a (Pheo-a), a photosensitizer derived from chlorophyll-a, to either chemically modified lyso-phosphatidylcholine (PhLPC) or egg lyso-sphingomyelin (PhLSM) were investigated to understand the impact of the lipid backbone of these conjugates on their self-assembly, as well as their physicochemical properties, including interfacial behavior at the air–buffer interface, fluorescence and absorption properties, thermotropic behavior, and incorporation rate in the membrane of liposomes [171]. XRR demonstrated that both lipid-porphyrin conjugates could be efficiently incorporated into lipid vesicles, with higher loading rates than unconjugated Pheo-a, as illustrated in Figure 19.

Nanoparticle toxicity to functioning of living cells is a topic of growing importance. In situ and real-time GIXF and GID study of the structural rearrangement in a model membrane (an arachidic acid monolayer) on a colloidal solution of cerium dioxide or magnetite showed that the character of the interaction of nanoparticles with the monolayer is determined by their chemical nature and size [172]. The interaction of membrane surfaces composed of lipids and lipopolymers was quantified at controlled dehydrating pressures using GIXF [173]. This technique yielded specific density profiles of the chemical elements P and S belonging to lipid headgroups and polymer chains, as well as counterion profiles for charged surfaces.

Nonionic dendritic amphiphiles that self-assemble into well-defined supramolecular aggregates are useful for the efficient solubilization of active agents, for example, in drug delivery. Dendritic amphiphiles based on a hydrophilic polyol dendron head connected to two-chain hydrophobic block behave analogous to phospholipids and form well-organized layers in bulk (vesicles) or at the water surface (Langmuir monolayer). Understanding the structure of mixed lipid membranes is important for the preparation of artificial membranes and vesicles with adjustable properties suitable for drug delivery applications. Phase behavior and microscopic structures of mixed Langmuir layers of these dendritic amphiphiles with well-known phospholipid DPPC were characterized by GID along the surface-area isotherm [174]. It was found that the dendritic generation and, by this, the headgroup size of the amphiphilic molecules have a significant influence on their interaction with DPPC at the air–water interface. Interaction between behenic acid (BA) spread on aqueous solution of chitosan was studied by XRR at different surface pressures of BA, mimicking the cell membrane, pH, and molecular weight of the polysaccharide [175]. The thickness of the polysaccharide layer derived confirmed that chitosan adsorbs flat at the interface at a specific range of pH. Numerous approaches exist for the development of new drug formulations aiming for advanced dissolution properties, including modern techniques like the preparation of water soluble inclusion complexes, self-(micro)emulsifying drug delivery systems, solid dispersions and solutions, nanosuspensions, and nano-extrusion. However, these approaches frequently turn out to be highly complex and hard to control on a large scale. A model drug phenytoin, 5,5-diphenyl-2,4-imidazolidinedione, which is applicable in various fields, as having anticonvulsive, antiepileptic, and antiarrhythmic effects in the human organism, was solution-processed onto SiO${}_{2}$ surfaces, and the films were investigated by a combination of XRR, GID, and AFM [176]. By altering the preparation parameters, a new surface-induced polymorph of the model drug was found.

### 4.3. Liquid Interfaces

Many natural processes take place at interfaces, e.g., a liquid–liquid interface, can adsorb various molecules and facilitate reactions such as growth of nanoparticles that can further self-assemble at the interface and form ordered structures [177]. Interfacial properties can be tailored by amphiphilic molecules that form monolayers [178] and an electric field can be used to drive ions from one fluid to another [179]. The XRR has been pivotal for the molecular scale understanding of liquid–liquid interfaces [6,177]. Selective extraction of metal ions from a complex aqueous mixture into an organic phase by means of amphiphilic carriers is used nowadays to separate toxic and radioactive metals from polluted environments and nuclear wastes. The molecular mechanism of the ion transport from an aqueous to an organic phase taking place at an aqueous–organic interface has been studied by a combination of XRR with neutron reflectivity [180]. It was shown that hard trivalent cations can be repelled or attracted by the extractant-enriched interface according to the nature of the ligand [181]. The process of metal ion extraction starts with hydration of ions in the aqueous phase, which are then transformed at the aqueous–organic interface into supramolecular complexes in the form of an inverted bilayer that dissolves in the oil phase. Surface layering at the mercury–electrolyte interface is another topic that has received considerable attention using high-resolution XRR [179]. The work demonstrated an ion-specific accumulation of anions and cations at the mercury–electrolyte interface. The high surface tension and electron density of the liquid mercury allow XRR measurements on a mercury surface to high *q* values and reach atomic-scale resolution.

Ionic liquids and deep eutectic solvents composed of two ionic components are of topical interest as green-chemicals, alternative solvents for amphiphilic self-assembly, electrochemistry, catalysis, etc. XRR study of mercury supported Langmuir films of imidazolium-based ionic liquids showed a gradual penetration of mercury into the ionic liquid film over several hours [182]. Surface-induced smectic order was found for the ionic liquid 1-methyl-3-docosylimidazolium bis(trifluoromethlysulfonyl) imide by XRR and GID experiments [183]. An ordered structure of alternating layers composed of polar and nonpolar moieties was observed near the free liquid surface that persists up to 88 K above the bulk melting temperature. Identical results were obtained with similar type of RTIL exhibiting liquid crystal bulk phases (1-alkyl-3-methylimidazolium bis(trifluoromethylsulfonyl)imides [C${}_{22}$mim]${}^{+}$[NTf2]${}^{-}$) [184]. Measured thickness of the liquid crystal at the interface confirmed the theoretically predicted logarithmic temperature dependence. GID measurements uncovered a 2 nm thick, hexagonally packed, crystalline monolayer formed at the sample surface. Generality of this behavior of RTIL [C${}_{n}$mim]${}^{+}$[NTf2]${}^{-}$ was confirmed by XRR and GID study of an homologous series with *n* varied from 4 to 22 [185]. Layering of cations and anions of RTIL in the vicinity of an interface is common for different type of liquids. This layering of electrolyte molecules gradually decays towards the bulk and the layering periodicity increases with the salt concentration. Indeed, the electrolyte ordering near a solid surface is of vital importance in physical chemistry, energy storage, and heterogeneous catalysis. e.g., a detailed picture of the electrode–electrolyte interface is relevant to Li-ion batteries [186]. The GIXF in combination with XRR can resolve the distribution of individual elements. This enabled to reveal the unequal partitioning of hydrophilic cations and anions at the liquid–vapor interface in a critical binary mixture of water and 2,6-dimethylpyridine containing KCl below its lower critical temperature [187,188]. The results with a resolution of one excess ion per 200 nm${}^{2}$ or better provided an unambiguous experimental evidence for microscopic segregation of hydrophilic ions in aqueous critical binary mixtures.

**Figure 20:**
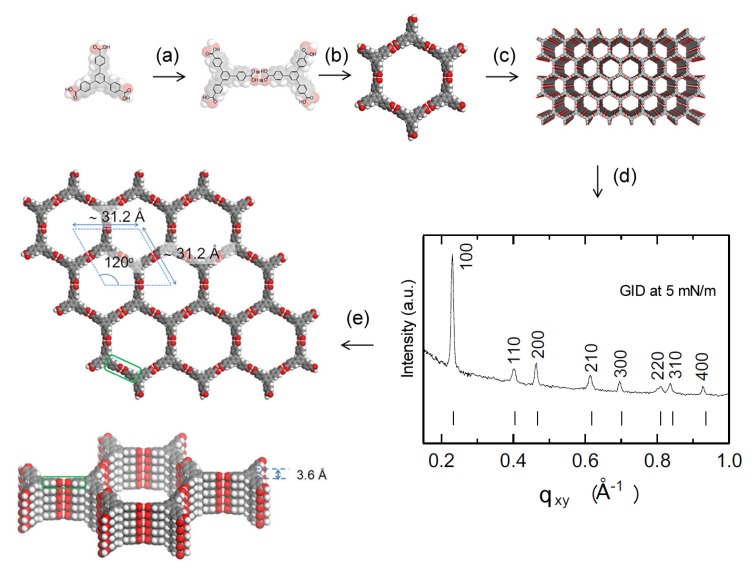
Formation stages of perfectly oriented nanosheets, out of building blocks of (1,3,5-tris-(4-carboxyphenyl)-benzene), with long-range molecular columns, and size- and position-regulated nanopores on an aqueous surface (**a**–**c**); (**d**) typical GID profile with $q_{xy}=\sqrt{q_{x}^{2}+q_{y}^{2}}$; and (**e**) reconstructed structure of the 2D crystal. Adapted from Makiura et al. [189] with permission from the American Chemical Society under ACS Author Choice License.

Nanosheets–free standing 2DM–with long-range structural order have been attracting considerable attention because of their intrinsic properties derived from their low dimensionality and their potential as ultrathin components in nanoelectronic devices, molecular separation and detection, catalysis, and energy conversion and storage. Incorporation of highly regulated nanopores into the 2D structure leads to enormous additional capacity in diverse applications. However, the availability of nanosheets has been hitherto restricted to layered parent materials, covalently bonded sheets, which are layered via relatively weak electrostatic interactions. A rational bottom-up methodology that enables nanosheet fabrication beyond the layered systems has been demonstrated with a triphenylbenzene derivative at the liquid–vapor interface under ambient conditions. The GID revealed the formation of a perfectly oriented highly crystalline noncovalent-bonded organic nanosheets [189]. Each molecular building unit connects laterally by hydrogen bonding, endowing the nanosheets with size and position regulated permanent nanoporosity as depicted in Figure 20. The synthesis of large scale 2DM on aqueous surface with long-range order (periodicity, crystallinity, etc.) out of 2D polymers is of significant interest. The behavior of compound 2 [1,3,5-tri(2,2${}^{\prime }$-bipyridin-5-yl)benzene] with three bipyridine units arranged in a star geometry was investigated in the presence and absence of Ni(ClO${}_{4}{)}_{2}$ by XRR [190]. It was found that the compound 2 stays almost vertical at the interface and at high Ni${}^{2+}/2$ ratios, two of the three bipyridine units are complexed, resulting in supramolecular sheets that are likely composed of arrays of linear metal-organic complex of the polymer.

The 2DM have already found numerous applications in materials science, and recently, a new family of 2DM, the so-called MXenes were discovered. Most MXene compounds exhibit metallic conductivity, hydrophilicity, and high flexibility. Unique combination of these properties makes MXene coatings significantly cheaper compared to graphene layers formed by conventional chemical vapor deposition. At a liquid–vapor interface, Ti${}_{3}$C${}_{2}$T${}_{x}$ MXene flakes spontaneously assemble into monolayer films. An XRR and GIXF investigation showed that both the structure of the layers and assembly kinetics depend on the pH of the solution [191]. Graphene oxide is another type of 2DM with increasing interest as a precursor for graphene based materials and as sensors, batteries, etc. These sheets adsorb to the air–water interface forming films and the characterization by XRR and GID elucidated that at non-zero surface pressures, the film is organized as a bilayer of sheets interfaced between air and water with water molecular bridges [192]. These results should allow precise control of the density of sheets deposited by the LB or Langmuir–Schaefer (LS) procedures. Graphene oxide is also extremely attractive for air and natural gas dehumidification and an operando study of ultrathin (50 nm) membranes by GID during air dehumidification has been reported [193]. It was found that the absorption of water vapor in graphene oxide layers follows a modified Kelvin equation similar to condensation in an elastic slit, whereas desorption of water is limited by a few outer layers. The interlayer distance depends on the partial pressure of water like in a feed stream and permeate [194]. Memristive elements are non-volatile electronic components with prospective application as artificial synapses, components of perceptrons, and more complex neuromorphic networks. Langmuir and LB/LS techniques allowed assembling model memristive elements using polymers polyaniline (PANI) and poly(ethylene oxide) (PEO) on planar thin films. The structure of planar conductive PANI-based materials were investigated by XRR and GID on liquid and solid surfaces depending on the PANI molar mass, nature of the solvent and sub-phase on the crystalline structure, thickness and conductivity of planar LB films, and the PANI/PEO ratio [195].

The increase in brilliance of the synchrotron sources, higher efficiency of detectors and better computing power allowed revisiting XRR studies of several longstanding questions on solid–fluid interfaces. For example, a study of naturally oxidized silicon surface immersed in water and covered by self-assembled monolayers (SAM) of octadecyltrichlorosilane revealed a very thin layer of low density in between the Si-substrate and SiO${}_{2}$ interface [196]. The angstrom-resolution of XRR and GID study of the structure of n-alkyltrichlorosilane (with n = 12, 14, 18, and 22) SAM on the amorphous native oxide of Si(100) showed that the alkyl chain packing order changes along its long axis in such a way that a moiety of molecules on the substrate side have higher crystallinity as compared to the part facing towards the gas media [197]. GID employed to probe the structure of atomically thin carbon layers (a monolayer and a bilayer graphene) on SiC(0001): the so-called buffer layer, demonstrated that it possesses a different lattice parameter, corrugation, and strain on the graphene layer [198].

### 4.4. Polymer Films

**Figure 21:**
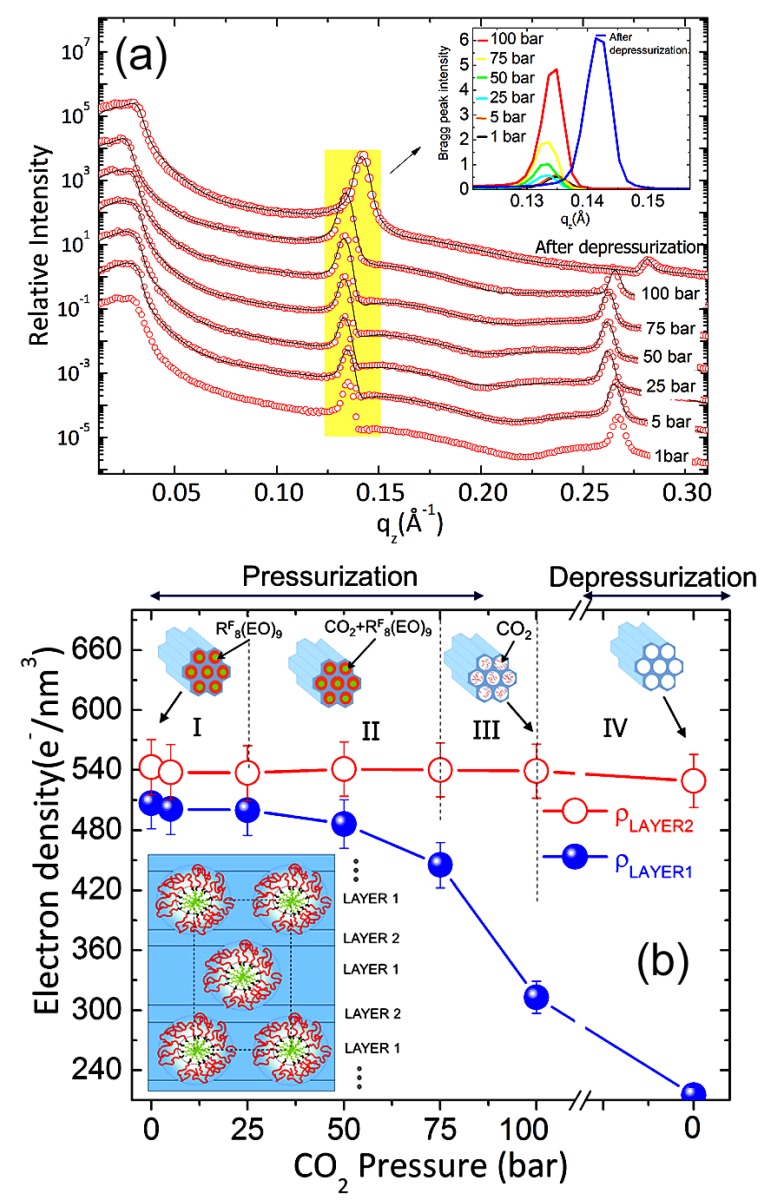
(**a**) Evolution of XRR profiles during in situ pressurization of CO${}_{2}$ in the cell. Curves are translated vertically for clarity (logarithmic scale). In the top inset, a zoom of the evolution of the intensity is shown in a linear scale. (**b**) Evolution of the total electron density of the layer 1 and layer 2 during the pressurization and after the depressurization. Adapted from Chavez Panduro et al. [201] with permission from the American Chemical Society under ACS Author Choice License.

The structure and properties of polymer thin films can be quite different from the properties of bulk polymers. For example, the glass transition temperature is often reduced in thin films as a consequence of the formation of a more mobile surface layer. Thin and ultrathin polymer films, with thicknesses comparable to one or a few polymer chains, also serve as models for studying the dynamical and structural properties of nanoscopically confined soft matter. XRR is ideal for probing the structure of buried polymer interfaces and their relation to the film properties. An XRR study of supported polystyrene thin films as a function of thickness and temperature [199] demonstrated an increase in the density and a decrease in glass transition temperature with thinning of the film. XRR study of polystyrene thin films isothermally exposed to CO${}_{2}$ as a function of pressure, starting from ambient pressure up to the supercritical state of CO${}_{2}$, revealed a swelling of the film and CO${}_{2}$ trapping at different stages of the pressure cycle [200]. High penetrability of supercritical CO${}_{2}$ and its efficiency as solvent were also found by XRR and GISAXS study of nonionic fluorinated surfactant removal from mesoporous films [201], as depicted in Figure 21. The removal of the fluorinated surfactant did not effect the lateral organization of the pores but led to shrinking of the film thickness, via reduction of the spacing between the rod-like pores.

Nanostructured polymer thin and ultrathin films have recently emerged as model systems to study the soft matter behavior under nano-confinement as well as promising active materials in a number of applications such as organic electronics, photovoltaics, biosensing, and biomaterials. In most of these cases, it is of paramount importance to control the film nanostructure, as well as the polymer conformation, to clearly define the confinement condition of the polymer and thereby optimize the device properties. Most common approaches to attain this goal are by exploiting the self-assembly properties of the polymer itself, tuning the interactions with the substrate to adapt polymer orientation, or applying the so-called top-down techniques. However, it is generally possible to control the film nanostructure only towards one direction with respect to the surface, that is, in either parallel or perpendicular, and only in special cases, mainly involving block copolymers, a full 3D control is attainable. A novel approach enabling the full control of the 3D nanostructure of P3HT thin films is realized using a nanoparticle monolayer as a template [202]. An ordered nanoporous film of P3HT is obtained upon etching the nanoparticles. The pores are then filled with a second organic component, PCBM, via spin coating. By modulating the deposition parameters enabled the control over the degree of filling with nanometric precision which was monitored by GISAXS measurements at each step of the film formation.

The photo-orientation process is a powerful and effective tool for the local manipulation of molecular orientation and controlling the optical properties of photochromic compounds. The origin of this process is associated with the selective polarized light excitation of the chromophores aligned along the polarization plane. The effects of molecular and supramolecular structure, thermal prehistory of the bent-shaped azobenzene-containing thin films, wavelength of the excitation light on the photo-optical properties, photo-orientation processes, and films morphology were revealed using GID [203,204]. It was shown that UV-irradiation leads to *E*-*Z* isomerization of azobenzene fragments in both amorphous and crystalline films of the synthesized compounds. This process is partially suppressed in crystalline films; nevertheless, UV-irradiation of the bichromophoric compounds resulted in a transition from crystalline to amorphous state and decrease in the surface roughness. Irradiation of amorphous films with polarized visible and UV light induced the photo-orientation of chromophores in a direction perpendicular to the polarization plane of the incident light. Observed photo-induced isothermal melting and photo-orientation processes could be exploited for the photo-optical data recording and storage.

Molecular self-assembly typically involving non-covalent interactions can be used as a bottom-up approach towards nanostructured functional materials. Supramolecular assemblies exhibit a higher sensitivity to external stimuli such as temperature, solvent, and magnetic and electric fields due to the intrinsic lability offered by the weak bonding. As a result, their properties can be fine-tuned to suit a wide range of applications without laborious chemical modification of molecular components. The thermotropic and lyotropic liquid crystalline phases formed by wedge-shaped amphiphiles have great potential for practical applications such as controlled delivery. Particular attention has been recently focused on bicontinuous liquid crystalline cubic phases. These phases are characterized by a macroscopic channel continuity even in the absence of orientation, which makes them interesting for proton conduction. Using room temperature GIWAXS, for the first time, a mechanism for physical activation of the alkyl-substituted wedge-shaped sulfonate molecule (C8Pyr) films, involving a transformation of a monoclinic columnar structure to a bicontinuous cubic phase, was revealed [205].

### 4.5. Block Copolymer Nanostructures

**Figure 22:**
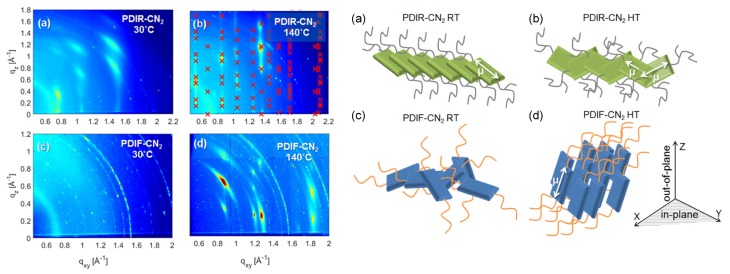
Schematic representation of microdomain reorientation during swelling of a block copolymer thin film in a non-selective solvent (chloroform) vapors together with the GISAXS pattern at the initial (1) and the final states (3). Phase diagram in the center of the bottom raw shows the pathway during the swelling-drying procedure. Adapted from Gowd et al. [206] with permission from the Royal Society of Chemistry ©2014.

The ability of block copolymers to spontaneously self-assemble to well-ordered arrays of microdomains with a typical spatial period of a few tens of nanometers make them attractive for many applications. GISAXS is among the principal techniques to characterize these structures, essential for fine-tuning their properties and obtaining the desired morphology. For example, an in situ GISAXS study of solvent vapor annealing revealed the orientation changes of microdomains in polystyrene-block-poly(4-vinylpyridine) (PS-b-P4VP) thin films [206]. The swelling of P4VP perpendicular cylinders in chloroform, a non-selective solvent vapor, led to the reorientation of cylinders to in-plane configuration through a disordered state along a particular kinetic pathway in the phase diagram as shown in Figure 22. On the other hand, the swelling of the P4VP perpendicular cylinders in a block selective solvent vapor (1,4-dioxane) induced a morphological transition from cylindrical to spherical microdomains via an ellipsoidal transient structure. The solvent evaporation resulted in shrinkage of the matrix in the vertical direction, merging the ellipsoidal domains to form perpendicularly aligned cylinders.

A promising class of ultrathin films is Langmuir monolayers of amphiphilic block copolymers, as they can self-assemble into unique microphase-separated 2D nanostructures by simply tuning parameters such as surface pressure and block length ratio [207]. 2D self-assembly at the air–water interface can also be used to tune polymer–polymer interactions at the interface and the morphology of the film that can be subsequently transferred onto a solid substrate. PS-b-PAA block copolymer monolayers self-organized at an air–water interface can reversibly expel the remaining solvent, DMF, by cyclic variation of lateral compression [208,209]. GID peaks from block copolymer changed intensity depending on the surface pressure applied to the monolayer. GIWAXS measurements revealed unprecedented details on the crystallization of PEO during the compression of Langmuir films on a 4 molar aqueous solution of K${}_{2}$CO${}_{3}$, which is a kosmotropic salt according to the Hofmeister series [210]. The initial pancake structure that is flat-lying PEO molecules adsorbed at the liquid–vapor interface had low surface coverage, identical to the behavior of PEO on neat water. With the onset of desorption from the interface during compression, the PEO started to entangle and formed a 3–4 nm thick amorphous layer. The PEO layer crystallized with further compression, and the crystal orientation was observed by GIWAXS.

### 4.6. Polymer Photovoltaics

Semiconducting polymer films and polymer blends form the basis of modern plastic electronics such as organic solar cells, light-emitting diodes (LED), field effect transistors (FET), as well as biosensors, which are mechanically flexible and lightweight materials. Organic photovoltaics (OPV) is one of the most promising technologies for sustainable green energy supply. However, to exploit their great potential, further improvements in their device performance are required. In situ X-ray scattering techniques are powerful to investigate these materials whose performance depends critically on their micro- and nanostructure at buried interfaces [211,212], as the charge carriers are located within the first monolayer of molecules in close proximity to the dielectric interface. The charge transport in organic thin film is directly related to the orientation, the domain size and the efficient interaction of π-stacked organic semiconducting molecules. The XRR is an ideal tool for establishing a detailed understanding of the vertical structure of conjugated-polymer fullerene thin films in bulk-heterojunction and OPV. In spite of the low X-ray scattering contrast between different polymers and organic materials, the obtained SLD profile between the polymer matrix and the fullerene allows the determination of the distribution of components and correlate it with the electronic properties. For example, the use of growth interruptions and the deposition rate to manipulate the phase separation mechanism [213]; the correlation and balance between the miscibility and optoelectronic properties of polymer–fullerene solar cells [214]; the formation of SAM of functionalized fluorinated alkyl fullerenes on aluminum oxide surfaces [215]; the development of unidimensional fullerene channels along the π-stacking direction of the polymer crystallites during doctor blading with subsequent controlled polymer drying [216]; and the alignment of the $C_{60}$ head groups within the SAM tuned by the alkyl phosphonic acids chain length [217], etc. In situ measurements also revealed a previously unknown mechanism of how additives can influence the polymer: fullerene bulk-heterojunction microstructure formation [218].

**Figure 23:**
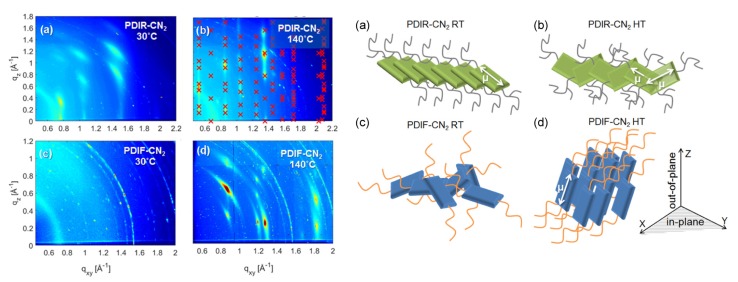
Schematic representations of molecular arrangements in (**a**) PDIR-CN2 RT-phase, (**b**) PDIR-CN2 HT-phase, (**c**) amorphous PDIF-CN2, and (**d**) PDIF-CN2 thin film crystal phases together with corresponding GID measurements, where $q_{xy}=\sqrt{q_{x}^{2}+q_{y}^{2}}$. Adapted from Belova et al. [219] with permission from the American Chemical Society under ACS Author Choice License.

Organic semiconductors (OSC) as active layers in OPV devices, and organic FET and LED provide not only benefits such as high efficiency, low costs, processability, and device flexibility, but also enormous possibilities for tuning their optoelectronic properties in a controlled way by changing their chemical composition and structural organization. The GID in combination with XRR provide the structural information both in-plane and along the surface normal. Among different types of organic donors (p-type polymers) and organic acceptors (n-type polymers), perylene diimide (PDI) derivatives represent a well-known family of electron-accepting small molecule OSC. Hydrogenated PDIR-CN2 and fluorinated PDIF-CN2 version of PDI molecules deposited with organic molecular beam deposition method on top of native silicon oxide demonstrated an increase of lateral and transversal crystallinity upon heating the film from room temperature to 110–160 °C, whereas PDIF-CN2 is poorly ordered at room temperature right after deposition [219] as shown in Figure 23. The charge transfer between electron donor and electron acceptor molecules at interfaces or in mixed molecular systems is one of the fundamental processes in devices based on OSC. The charge transfer strongly depends on the molecular composition, mixing ratio and the structural architecture. A systematic XRR and GID study of the charge transfer mechanism in a two-component system of diindenoperylene (DIP) and PDIR-CN2 with different architectures, namely a mixed bulk-heterojunction film at various concentrations, a planar-heterojunction and a superlattice, showed [220] that the strong intermolecular coupling together with the intimately mixed structure without phase separation contribute to the large energy losses in solar cells of these materials. Effects of the side chains and thermal annealing of the OSC films on their electrical characteristics in organic FET have been explored using a series of eight substituted PDI [221]. Clear correlations between the thermally-induced improvement in the crystallinity of PDI films evidenced by GIWAXS, their phase transition temperatures and the electrical characteristics of the organic FET have been revealed. Determination of the molecular orientation on a solid substrate is especially important in organic (opto)electronics, where the properties such as the charge-carrier lifetime, interfacial energetics, or the light absorption are strongly correlated with the anisotropic nature of molecules. GIWAXS study of thin DIP film on MoS${}_{2}$ showed that DIP forms separate islands on the top of the MoS${}_{2}$ monolayer with lying-down orientation of the molecules in a triclinic lattice [222]. GID study of planar-heterojunctions of molecular donor material α-sexithiophene with DIP acceptor molecules described structural difference, crystallinity, and the molecular orientation, in films grown at room and 100 °C [223] and linked them with the characteristic parameters of the corresponding OPV cells.

Efficiency of OPV cells comprising a mixtures of two organic compounds crucially depends on the extent of phase separation of the electron donating and electron accepting materials, particularly in comparison to the length-scales of the electronic processes (e.g., exciton diffusion length) in the system. A comprehensive study of all important parameters that can be used to tailor the extent of phase separation in OSC mixtures was performed in a 1:1 blend of DIP and buckminster fullerene (C60) grown by molecular beam deposition at different substrate temperatures, different growth rates, time-dependent deposition rates, and surface functionalization layers [224]. The same question was addressed by combining GID and XRR to study the growth kinetics of ultrathin films of a prototypical OSC DIP [225]. The identical character of the substrate temperature for growth of OPV and organic LED and FET from small-molecule OSC was demonstrated by growth, structure, and anisotropic optical properties of difluoroanthradithiophene thin films [226]. The prototypical n-type OSC PDIF-CN2 was assembled into well-ordered nanoarchitectures by LB technique and a multiscale analysis of the correlation between their structural and electrical properties was performed using XRR and GID [227]. LB-deposited monolayers of PDIF-CN2 are arranged in upright standing molecular packing on different substrates. Post-deposition thermal treatment leads to a reorganization into layered ultrathin crystalline nanostructures, exhibiting structural and photophysical properties similar to those of microscopic crystals obtained by solvent-induced precipitation. The commonality of the influence of surface energies and polarities of the substrate surfaces on the organization of organic molecules has also been reported [228].

A real-time simultaneous GISAXS and GIWAXS study of pentacene growth on epitaxial graphene on silicon carbide with millisecond time resolution identified two distinct anisotropic growth stages after the nucleation of the first monolayer [229]. Another in situ investigation of the growth behavior of pentacene within thin films as a function of film thickness ranging from 20 to 300 nm provided a better insight into the transition from the metastable thin film phase to bulk phase polymorphs [230]. Despite the fact that both pentacene and its isomer picene being planar π-conjugated compounds, their electronic properties are very different in thin films as evidenced by a real-time XRR and GID study [231]. Blends of OSC are relevant for numerous electronic and optoelectronic applications, but their properties in thin films strongly depend on the morphology, crystallinity, and degree of intermixing. There are several factors contributing to these effects, most notably the intermolecular interaction energies of combinatorial pairs and the steric compatibility, i.e., the relative size and shape of individual components. These aspects of binary OSC mixtures of picene (C22H14) and perfluoropentacene (C22F14) as well as picene and pentacene, simultaneously coevaporated on substrates onto thin films were systematically investigated using GID, AFM, and X-ray absorption spectroscopy [232]. The OSC doping is an effective method for tuning the ground-state charge transfer, and, in this case, X-ray scattering techniques are useful for obtaining an understanding of the link between the electronic properties and structural organization of co-evaporated polymers on molecular scale. Although the doping-induced relative increase in conductivity appears comparable between P3HT, the benchmark polymer, and quaterthiophene films, the charge transfer scenario found for P3HT does not occur for p-doped quaterthiophene, despite their similar microstructure [233].

Hybrid organic–inorganic perovskites are another promising material for OPV possessing high efficiencies, which can be created by mixing different halides or by mixing different organic cations. The use of multiple halides or cations can help to tune the band gaps and improve the stability of perovskite films. Furthermore, the addition of chloride or bromide to methylammonium lead iodide (MAPbI${}_{3}$) has been shown to improve the transport and diffusion lengths of charge carriers. The exchange of ions in hybrid organic–inorganic perovskites with the general formula APb${}_{3}$ (A = MA, FA; X = I, Cl, Br) was studied in five different systems using in situ real-time GID [234]. In systems where the organic cation is exchanged, a continuous shift of the lattice parameter was found and used that to quantify the ion exchange. GIWAXS study of the inorganic CsPbI${}_{3}$ planar junction perovskite solar cells allowed to find an optimal structure for photovoltaic performance among different temperature-dependent phases [235]. Perovskite solar cells with CuSCN hole extraction layers yield stable efficiencies greater than 20%. This is associated with a preferential out-of-plane orientation of CuSCN domains with the long unit cell axis parallel to the substrate as observed in GIWAXS patterns [236].

Benzothieno[3,2-b][1]benzothiophene (BTBT) is another organic p-type semiconductor which forms 2D SAM with π-staking. XRR and GID study of BTBT linked to a C11 or C12 alkylphosphonic acid revealed an excellent correlation of structural morphologies with predictions by molecular dynamics simulations and semiempirical molecular-orbital electronic-structure calculations [237]. Films with C11 showed better crystallinity and improved charge transfer thanks to the larger domain size and the lower tilt angle of the BTBT functional end groups in BTBT-C11-PA SAM. Monomolecular layers of BTBT organosilicon dimer D2-Und-BTBT-Hex, prepared on the water surface and transferred on to solid substrates, also showed better electronic properties of fabricated FET devices with increase in the crystallinity of the film [238].

In the examples presented above, the OSC films were prepared using molecular beam deposition, LB, LS, or spin-coating methods. The blade-coating is another widely used way to prepare OSC from polymer blend solutions. The final microstructure of a polymer bulk-heterojunction film is a fine interplay between the solution thermodynamics and kinetics during the drying process. The use of solvent additive has become a successful strategy to control the structural evolution upon film formation in solar cells [239]. The specific role of 1,8-octanedithiol additive on the crystallization directly after wet film deposition using blade-coating was studied from the earliest time of solvent evaporation using in situ X-ray scattering at different blend concentrations [240]. Multiple charge transfer pathways in OSC films were identified by XRR and GID investigations of different π-motifs, with π-system shapes ranging from stick-like structures, to spherical shapes such as in fullerenes [241]. The structure-charge transfer correlation found in OSC polymer blends is also applicable for block copolymers, as demonstrated using a combination of X-ray scattering, AFM and vertical charge transport measurements in diode devices on thin films of donor–acceptor block copolymers [242].

## 5. Summary and Outlook

The examples presented in the previous sections clearly illustrate that X-ray scattering methods are very powerful to address a large number of issues in nanomaterials and soft matter science. As these materials are involved in modern technology and a large fraction of consumer products in everyday life, a deeper understanding of their fundamental physics and chemistry is important for the rational design and engineering of new functional materials with long-term sustainability. In addition to their importance in practical applications, soft matter and nanomaterials figure in the broader picture of complex systems as models for fundamental investigations. Indeed, these materials are very relevant to better understanding of many biological processes, and development of new methodologies in biophysical and medical research.

**Figure 24:**
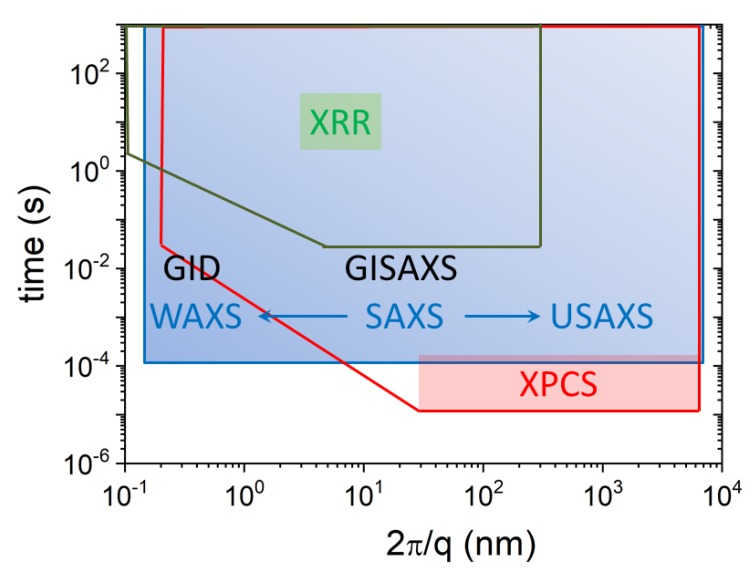
Nominal size and time scales accessible by different synchrotron X-ray scattering methods using 2D detectors. The exact range depends on the scattering features and contrast of the sample. For XPCS the smaller scales are limited by the longitudinal coherence, flux and detector resolution.

Although, direct imaging techniques are fast advancing, scattering methods remain relevant for investigations under in situ and real-life conditions. Furthermore, the ensemble averaged information is readily obtained, which is important for predicting the macroscopic behavior on the basis of microstructural models. The key strength of synchrotron radiation is the high brilliance which implies high flux, low divergence, and small beam size. In combination with advanced detectors, this translates to high angular, spatial, and temporal resolutions in the investigation of microstructure and dynamics. Figure 24 summarizes the nominal length and time scales accessible by real-time X-ray scattering methods at synchrotron sources. High energy X-rays (>20 keV) have a clear advantage for studying buried interfaces due to their high penetration power. Micro/nanobeams allow exploration of small specimens and scanning experiments with high spatial resolution, which can then decipher larger scale microstructures in real space. However, a serious disadvantage of high brilliance X-rays is the impingent radiation damage which is inherent with soft materials, especially that most studies need to be performed under the appropriate thermodynamic conditions. However, the rapid advances in detector technology together with optimized data acquisition strategies help to overcome this limitation and obtain good data prior to the onset of radiation damage in most cases.

The new generation synchrotrons based on multi-bend achromat storage ring lattices (such as the ESRF EBS, APS-U, etc.) are expected to enhance the brightness by at least two orders of magnitude, whereas XFELs have 4–6 orders of magnitude higher peak brightness as compared to the third generation synchrotrons. The broad range of issues in nanomaterials and soft matter may not justify the access to XFEL instruments. As a result, synchrotron facilities are likely to remain at the center stage for X-ray investigations of these materials. The high degree of coherence of the upcoming sources would close the gap between XPCS and dynamics accessible by quasi-elastic neutron scattering. With extremely brilliant synchrotron sources, the coherent flux at high energies will be significantly higher that will enable probing the dynamics at buried interfaces by XPCS. The advances in X-ray instrumentation must be complemented by development of new sample environments for in situ and complex conditions. Advanced modeling and computer simulations are indispensable for deriving quantitative structural and dynamical information from scattering experiments. In this respect, the machine learning techniques are likely to be more exploited in the analysis of scattering data in the future [243,244]. The ultimate goal is to derive the real space images of functional systems with nanometer scale resolution from the scattering data by exploiting the coherence and inherent features within the scattering patterns.

## Abbreviations

The following abbreviations are used in this manuscript:

2D	Two-dimensional
2DM	Two-dimensional materials
3D	Three-dimensional
AFM	Atomic force microscope
CDI	Coherent diffractive imaging
DMF	N,N-dimethylformamide
FET	field effect transistors
GI	Grazing incidence
GID	Grazing incidence diffraction
GISAXS	Grazing incidence small-angle X-ray scattering
GIWAXS	Grazing incidence wide-angle X-ray scattering
GIXF	Grazing incidence X-ray fluorescence
GIXPCS	Grazing incidence X-ray photon correlation spectroscopy
LB	Langmuir-Blodgett
LED	light-emitting diodes
LS	Langmuir–Schaefer
OPV	Organic photovoltaics
OSC	Organic semiconductors
RTIL	room temperature ionic liquids
SAM	self-assembled monolayers
SANS	Small-angle neutron scattering
SAXD	Small-angle X-ray diffraction
SAXS	Small-angle X-ray scattering
SLD	scattering length density
TR-SAXS	Time-resolved small-angle X-ray scattering
USAXS	Ultra small-angle X-ray scattering
WAXS	Wide-angle X-ray scattering
XFEL	X-ray free-electron laser
XPCS	X-ray photon correlation spectroscopy
XRR	X-ray reflectivity
